# Non-invasive assessment of murine PD-L1 levels in syngeneic tumor models by nuclear imaging with nanobody tracers

**DOI:** 10.18632/oncotarget.16708

**Published:** 2017-03-30

**Authors:** Katrijn Broos, Marleen Keyaerts, Quentin Lecocq, Dries Renmans, Tham Nguyen, David Escors, Adrian Liston, Geert Raes, Karine Breckpot, Nick Devoogdt

**Affiliations:** ^1^ Laboratory of Molecular and Cellular Therapy (LMCT), Vrije Universiteit Brussel, B-1090 Brussels, Belgium; ^2^ *In Vivo* Cellular and Molecular Imaging Laboratory (ICMI), Vrije Universiteit Brussel, B-1090 Brussels, Belgium; ^3^ Nuclear Medicine Department, UZ Brussel, B-1090 Brussels, Belgium; ^4^ Unit of Cellular and Molecular Immunology (CMIM), Vrije Universiteit Brussel, B-1050 Brussels, Belgium; ^5^ Myeloid Cell Immunology Laboratory, VIB Inflammation Research Center, 9052 Ghent, Belgium; ^6^ Immunomodulation Group, Navarrabiomed-Biomedical Research Centre, 31008 Pamplona, Navarra, Spain; ^7^ Department of Microbiology and Immunology, University of Leuven (KU Leuven), 3000 Leuven, Belgium; ^8^ VIB Center for Brain and Disease Research, 3000 Leuven, Belgium

**Keywords:** immune checkpoints, programmed death-1/programmed death-ligand 1, biomarker, nanobodies, SPECT/CT imaging

## Abstract

Blockade of the inhibitory PD-1/PD-L1 immune checkpoint axis is a promising cancer treatment. Nonetheless, a significant number of patients and malignancies do not respond to this therapy. To develop a screen for response to PD-1/PD-L1 inhibition, it is critical to develop a non-invasive tool to accurately assess dynamic immune checkpoint expression. Here we evaluated non-invasive SPECT/CT imaging of PD-L1 expression, in murine tumor models with varying PD-L1 expression, using high affinity PD-L1-specific nanobodies (Nbs). We generated and characterized 37 Nbs recognizing mouse PD-L1. Among those, four Nbs C3, C7, E2 and E4 were selected and evaluated for preclinical imaging of PD-L1 in syngeneic mice. We performed SPECT/CT imaging in wild type versus PD-L1 knock-out mice, using Technetium-99m (^99m^Tc) labeled Nbs. Nb C3 and E2 showed specific antigen binding and beneficial biodistribution. Through the use of CRISPR/Cas9 PD-L1 knock-out TC-1 lung epithelial cell lines, we demonstrate that SPECT/CT imaging using Nb C3 and E2 identifies PD-L1 expressing tumors, but not PD-L1 non-expressing tumors, thereby confirming the diagnostic potential of the selected Nbs. In conclusion, these data show that Nbs C3 and E2 can be used to non-invasively image PD-L1 levels in the tumor, with the strength of the signal correlating with PD-L1 levels. These findings warrant further research into the use of Nbs as a tool to image inhibitory signals in the tumor environment.

## INTRODUCTION

Cancer cells express neo-antigens generated due to mutations and aberrant processing of proteins as well as cancer-germline antigens generated due to epigenetic alterations. Consequently, CD8^+^ T cells can recognize them as non-self [1]. However, cancer cells co-opt specific inhibitory signaling pathways, known as immune checkpoints to evade their CD8^+^ T cell-mediated destruction [2]. Inhibition of these inhibitory immune checkpoints has received increasing interest as a disruptive treatment for patients with solid and hematological tumors [3–16].

Several inhibitory immune checkpoints, consisting of inhibitory receptors expressed on T cells and their ligands expressed on antigen-presenting cells have been described. Of these the immune checkpoint consisting of programmed death-1 (PD-1, CD279) and its ligand programmed death-ligand 1 (PD-L1, B7-H1, CD274), and its blockade with monoclonal antibodies (mAbs) have received much attention [7, 11, 15–19]. FDA approved mAbs targeting PD-1 include Nivolumab (Bristol-Myers Squibb) and Pembrolizumab (Merck), while Atezolizumab (Roche) is the first PD-L1 targeting mAb to be FDA approved [20].

Although treatment with antagonistic PD-1 and PD-L1 specific mAbs has shown encouraging results across different indications, a substantial number of patients do not respond. Therefore, there is a need to accurately predict which patients will benefit from this treatment. A number of correlative studies utilizing invasive biopsy in conjunction with immunohistochemistry (IHC) suggest that PD-1 expression on tumor-infiltrating CD8^+^ T cells could serve as a predictive marker [21]. Similarly, the use of PD-L1 expression in the tumor environment as a biomarker has been investigated. Indeed, across multiple cancer types, there is a strong positive correlation between pre-treatment PD-L1 expression (irrespective of its expression on tumor cells or infiltrating immune cells) and therapeutic response to PD-1/PD-L1 pathway inhibition [16]. Nonetheless, patients showing PD-1 or PD-L1 expression can fail anti-PD-1 or anti-PD-L1 mAb therapy, while patients that show no PD-L1 expression were reported to benefit from anti-PD-L1 mAb therapy [22]. This can be explained by the high heterogeneity of tumors and by the role that PD-L1 plays during antigen presentation to T cells by DCs [23, 24]. Both the expression of PD-1 and PD-L1 are highly heterogeneous within the primary tumor as well as in metastases. Moreover, the expression of PD-1 and PD-L1 are likely to change in time and can be influenced by factors in the microenvironment, such as IFN-γ secretion [25]. Consequently, the static picture of a biopsy is not ideal to predict therapy outcome. IHC as a technique has the additional limitations that it does not provide information about the PD-1/PD-L1 expression in all lesions, and that it can only be performed on tumors that are accessible for biopsy. Therefore, there is a compelling need to develop a non-invasive imaging strategy to determine the presence of immune checkpoints in cancer patients before and during the course of treatment.

Imaging of PD-L1 or PD-1 using radiolabeled mAbs has recently been reported [26–31]. However, mAbs have inherent limitations that can curtail their efficacy as an imaging tool. Because of their size, they show poor capacity to penetrate tissue (tumor) and because of their long circulation time, high-contrast imaging can only be done after multiple days and with a risk of false positive results due to the remaining blood pool activity, especially for targets with low levels of expression like PD-L1 [32]. Therefore, there is a need for an alternative antigen-binding moiety with improved traits as a tracer. For instance, recent studies reported on the utility of small, affinity-matured compounds derived from the PD-1 extracellular domain as tracers to visualize (human) PD-L1 in the tumor as early as one hour after injection [33]. Likewise, Nanobodies (Nbs), which are the smallest antigen binding fragments that can be obtained from unique camelid heavy-chain-only antibodies, are attractive alternatives to mAbs. Nbs are stable, soluble and have a high specificity and affinity [34]. Furthermore, Nbs efficiently enter tissues where they rapidly and specifically bind their antigens, while unbound Nbs are quickly cleared through renal elimination. Consequently, Nb tracers usually generate higher target-to-background signals and earlier after administration as compared to mAbs [32].

In the current study, we developed tracers for preclinical imaging of PD-L1 using Nbs radiolabeled with technetium-99m (^99m^Tc). To this end, a library of anti-PD-L1 Nbs was screened and the identified anti-PD-L1 Nbs were ranked according to affinity and specificity for human and/or mouse PD-L1. Four Nbs were selected and their biodistribution was evaluated in wild type and PD-L1 knock out mice using SPECT/CT. Based on these results, two lead compounds were chosen for imaging of PD-L1 in syngeneic tumors. These syngeneic mouse models enabled us to show that radiolabeled Nbs can visualize PD-L1 expression and that Nb accumulation correlates with levels of PD-L1 in the tumor, even when such PD-L1 expression was low. The latter reflects the clinical situation where the threshold for PD-L1 positivity by IHC is low (often only 5%) [4]. Taken together, these results confirm the high potential of nanobody-based PD-L1 imaging and substantiate the development of anti-human PD-L1 Nbs for clinical translation.

## RESULTS

### Generation and selection of Nbs recognizing PD-L1

**Figure 1:**
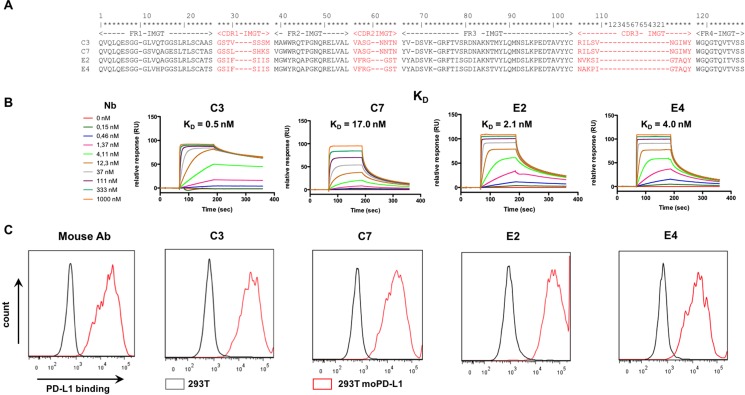
Selection of anti-mouse-PD-L1 specific Nbs (**A**) Amino acid sequence alignment of the purified Nbs C3, C7, E2 and E4. The Nb sequence includes three complementarity-determining regions (CDR 1, 2, 3; indicated in red) and four framework regions (FR1-4, indicated in black). FRs are relatively conserved but CDRs vary widely among Nbs. (**B**) Affinity/kinetics SPR study of purified Nbs interacting with immobilized His-tagged recombinant mouse PD-L1 protein. Sensorgrams of different concentrations of the Nbs are shown (*n* = 1). (**C**) Representative flow cytometry results, showing staining of unmodified HEK293T cells (grey line) or HEK293T cells lentivirally modified to express mouse PD-L1 (293T moPD-L1, red line) with mAbs specific for mouse PD-L1 or Nbs C3, C7, E4 and E2 (*n* = 3).

Nbs were raised against mouse PD-L1 by immunization of a dromedary with the PD-L1 expressing mouse macrophage cell line RAW264.7. Thirty-seven Nbs were selected after screening of an immune phage library on recombinant mouse PD-L1. Based on the amino acid sequence of the CDR1, 2 and 3 regions, these Nbs were divided into 9 sequence families (Supplementary Figure 1). Crude Nb-containing PEs were generated for further Nb characterization. The ability of the Nbs to bind mouse and human PD-L1 was determined in ELISA. The binding characteristics of the Nbs were compared using SPR measurements. Moreover, flow cytometry was performed to confirm binding to the mouse and human PD-L1 antigen when expressed on lentivirally modified B16 and HEK293T cells respectively (Supplementary Table 1). Nb families C and E bound particularly well on both mouse and human PD-L1 proteins with low background, and Nbs C3, C7, E2 and E4 were chosen for further evaluation (Figure 1A).

The selected anti-PD-L1 and control Nbs were then produced and purified as C-terminally His_6_-tagged proteins from E. coli fermentation cultures. Quality control confirmed good purity and low LPS content (Table 1). The affinity (K_D_) of the purified Nbs for the immobilized mouse PD-L1 antigen was determined using SPR (Figure 1B). The K_D_ values are in the low nanomolar range as shown in Figure 1 and Table 1. The ability of the different Nbs to bind to the mouse PD-L1 antigen when expressed on lentivirally modified HEK293T cells, was confirmed in flow cytometry (Figure 1C). We also used SPR to determine the affinity of the different Nbs for the human PD-L1 antigen. The affinity of the Nbs for the human PD-L1 antigen was substantially lower than for the mouse PD-L1 antigen (Supplementary Figure 2A). The ability of the different Nbs to bind to the human PD-L1 antigen when expressed on lentivirally modified HEK293T cells is shown in Supplementary Figure 2B.

**Table 1:** Summary of the endotoxin content, affinity for mouse PD-L1 and radiochemical purity of ^99m^Tc-Nb complexes of purified anti-PD-L1 Nbs C3, C7, E4 and E2

Nb	Endotoxins (EU/ml)	Affinity (KD)	Radiochemical purity
C3	< 50	0.5 nM	98.7
C7	< 50	17.0 nM	98.8
E2	59	2.1 nM	98.4
E4	67	4.0 nM	99.0

### Imaging of PD-L1 in naive mice

We radiolabeled the purified Nbs with ^99m^Tc through complexation of the ^99m^Tc-tricarbonyl with their His-tag. This is a site-specific labeling method with a low risk to interfere with their antigen binding capacity. Non-complexed ^99m^Tc was removed by gel filtration and aggregates present within the eluted ^99m^Tc-Nb preparation were removed by filtration. The radiochemical purity of the ^99m^Tc-labeled Nbs was assessed by iTLC measurement and was > 98% (Table 1).

**Figure 2:**
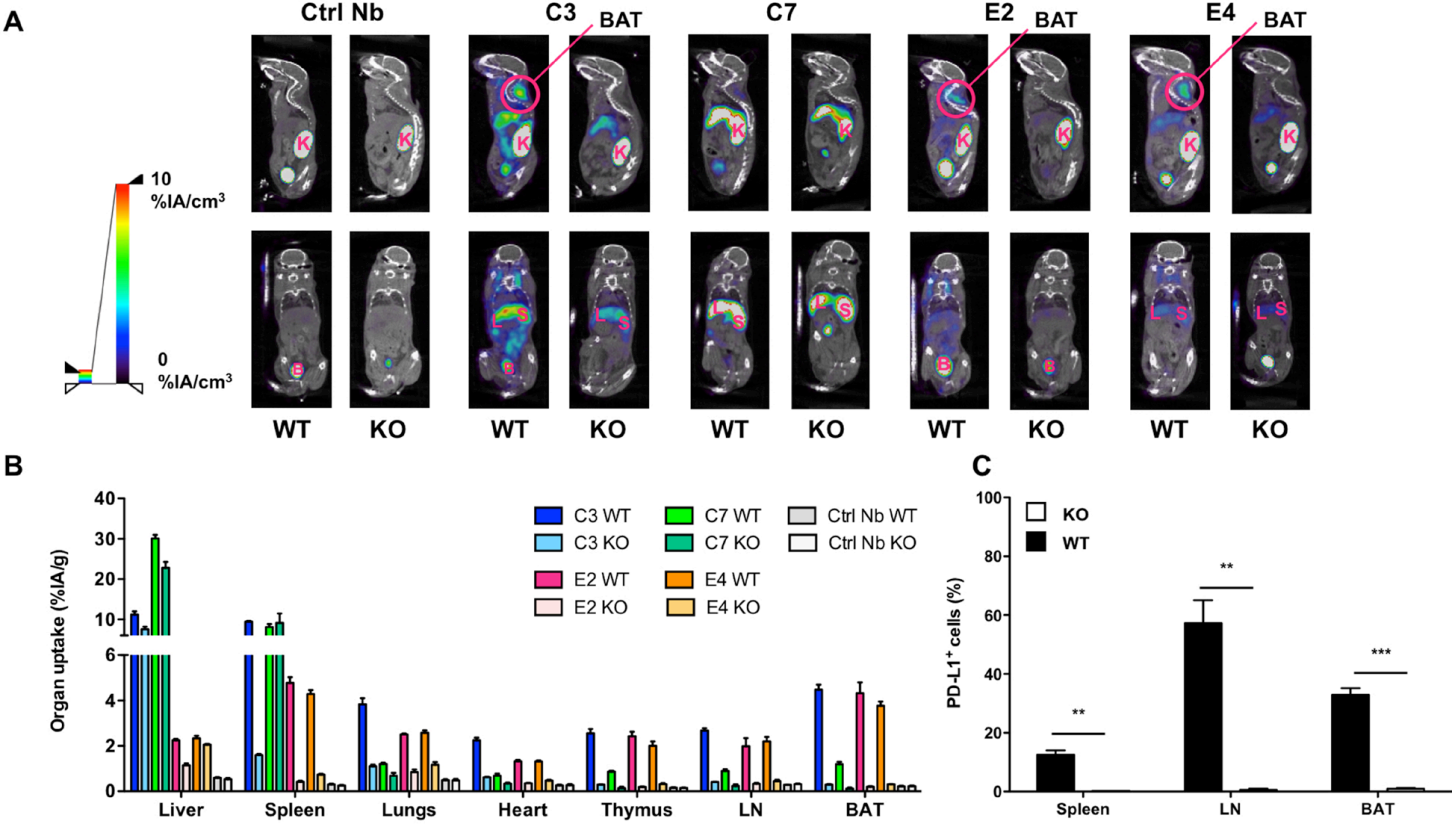
SPECT/CT and biodistribution evaluated 1 hour after injection of ^99m^Tc-Nbs C3, C7, E4 or E2 in naive wild type (WT) and PD-L1 knock out (KO) mice (**A**) Results of SPECT/CT scans to determine the biodistribution of ^99m^Tc-Nbs C3, C7, E4 or E2 injected in WT or PD-L1 KO mice (*n* = 3). (**B**) Gamma counting of isolated organs from WT or KO mice injected with ^99m^Tc-Nbs C3, C7, E4 or E2. The graph summarizes the organ uptake of the Nb per gram organ as the mean ± SEM (*n* = 3). (**C**) Percentage PD-L1 positive cells in spleen, lymph node (LN) and brown adipocyte tissue (BAT) of WT and PD-L1 KO mice using flow cytometry. The graph summarizes the percentage PD-L1 positive cells as mean ± SEM (*n* = 3). *K = kidney, L = liver, S = spleen, B = bladder, BAT = brown adipocyte tissue*.

To confirm their binding capacity and to distinguish specific from aspecific binding, the bio-distribution of each anti-PD-L1 Nb was compared in naive wild type and PD-L1 knock-out mice. SPECT/CT images taken 1 hour after intravenous tracer administration show high uptake in kidneys, bladder and to a certain extent also liver of wild type and knock-out mice (Figure 2A).

The high signals in kidneys and bladder are explained by the known kidney retention and urinary excretion of all Nbs. Specific organ uptake was observed, and was further evaluated using dissection and γ-counting (Figure 2B), confirming specific uptake for all Nbs in lungs, heart, thymus, spleen, lymph nodes and brown adipose tissue in high contrast to uptake of the Nbs in the blood. The γ-counting data of all organs and tissues that was determined after intravenous delivery of the ^99m^Tc-labeled Nbs is shown in Supplementary Table 2. Tissue versus blood, anti-PD-L1 Nb versus control Nb uptake ratios in wild type mice, and tissue uptake ratios in WT versus PD-L1-knock-out mice after intravenous administration of ^99m^Tc-labeled Nbs is shown in Supplementary Table 3. Nb C7 showed high and non-specific uptake in liver and spleen, making it less attractive as an imaging agent. C3, a family member to C7, also showed non-specific liver uptake, although to a lesser extent than C7. For the E-family members, the difference in organ uptake between wild type and PD-L1 knock-out mice is more pronounced for E2 than for E4.

Except for the accumulation of the Nbs in brown adipose tissue, we expected this biodistribution profile, because expression of PD-L1 on cardiac endothelium and cells of hematopoietic origin present within lung, spleen, thymus and lymph nodes has been well established [35–37]. However, the expression of PD-L1 in brown adipose tissue has only been recently described [31]. Therefore, we performed flow cytometry to analyze the expression of PD-L1 in brown adipose tissue, and compared it to PD-L1 expression on cells isolated from the spleen and lymph nodes. We observed that a high percentage of cells in these tissues expressed PD-L1 (Figure 2C).

### Detection of PD-L1 in tumor bearing mice

**Figure 3:**
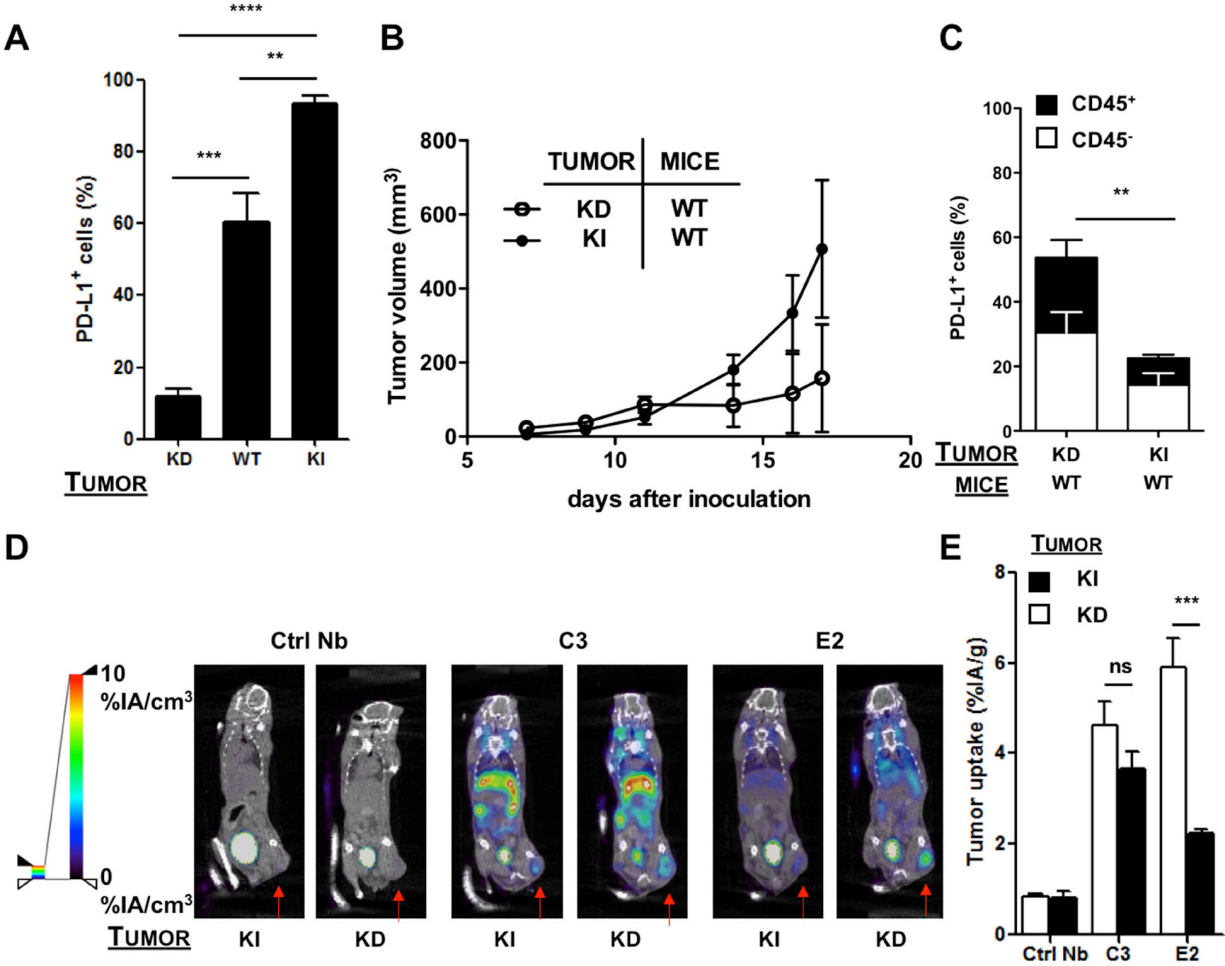
SPECT/CT in the shRNA-modified TC-1 model 1 hour after injection of ^99m^Tc-Nbs C3 or E2 (**A**) Percentage of PD-L1 on TC-1 cells transduced with lentiviral vectors harboring shRNA against mouse PD-L1 (knock-down, KD), wild type TC-1 cells (WT) or TC-1 cells transduced with mouse PD-L1 (knock-in, KI), evaluated with flow cytometry (*n* = 5). The graph summarizes the percentage PD-L1 positive cells as mean ± SEM. (**B**) TC-1 KD or KI cells were injected subcutaneously at the tail base of C57BL/6 mice. Tumor growth was followed every other day. The evolution of tumor size is shown as mean ± SEM (*n* = 5). (**C**) Mice were sacrificed on day 12 and tumors were isolated, after which expression of PD-L1 on tumor cells (CD45^−^, white bar) and tumor-infiltrating immune cells (CD45^+^, black bar) was evaluated in flow cytometry. The graph summarizes the percentage of PD-L1 as mean ± SEM (*n* = 5). (**D**) Images of SPECT/CT scans to determine the accumulation of ^99m^Tc-Nbs C3 and E2 in C57BL/6 mice bearing KI (left panel) or KD (right panel) tumors (*n* = 6). The red arrow indicates the tumor on the images. (**E**) Graphs showing the quantified *ex vivo* analysis to determine the accumulation of ^99m^Tc-Nbs C3 and E2 in PD-L1 KI (black bar) or KD (white bar) tumors. The graph shows the percentage radioactivity per gram tumor as mean ± SEM (*n* = 6).

To determine whether anti-PD-L1 Nbs are able to detect differences in PD-L1 expression in tumors, we set out to generate a model with low and high mouse PD-L1 expression. Hereto, we transduced TC-1 lung epithelial cells with lentiviral vectors that harbor the murine PD-L1 gene or a shRNA targeting mouse PD-L1 to generate a PD-L1 knock-in and knock-down TC-1 model respectively. Expression of PD-L1 on TC-1 wild type, knock-down and knock-in cells was evaluated by flow cytometry, showing < 20% to nearly 100% of cells expressing PD-L1 in the PD-L1 knock-down and knock-in TC-1 cells respectively (Figure 3A).

The PD-L1 knock-down and knock-in TC-1 cells were transplanted subcutaneously in wild type mice, and tumor growth was evaluated every other day. We observed a delayed outgrowth of TC-1 tumors in the PD-L1 knock-down model, highlighting the critical role of PD-L1 in tumor development (Figure 3B). We performed SPECT/CT imaging 1 hour after injection of the ^99m^Tc-labeled Nbs C3 and E2, followed by evaluation of accumulation of these Nbs in individual organs (*ex vivo* analysis). Against our expectations, *in vivo* and *ex vivo* analysis showed the highest accumulation of both Nbs in tumors grown from the PD-L1 knock-down TC-1 cells (Figure 3D–3E).

Dissection and biodistribution data obtained at 80 minutes after Nb injection using γ-counting is shown in Supplementary Table 4 (on the left), confirm these findings. Tissue-to-blood ratios of all organs derived from the biodistribution data are shown in Supplementary Table 5 (on the left). To further investigate the cell type being targeted, we isolated tumors grown from the PD-L1 knock-down and knock-in TC-1 cells and evaluated the expression of PD-L1 by flow cytometry. In accordance with the imaging data, flow cytometry showed a significantly higher expression of PD-L1 in tumors grown from the PD-L1 knock-down cells. This was observed on both immune (CD45^+^) and non-immune (CD45^−^) cells, suggesting that the shRNA expressed in these cells is inefficient in mediating substantial knock-down of PD-L1 *in vivo* and a compensatory upregulation of PD-L1 expression on immune infiltrates (Figure 3C). We hypothesize that initially the expression of PD-L1 is low in tumors of wild type mice inoculated with PD-L1 knock-down TC-1 cells, thereby allowing CD8^+^ T cells to interact with the tumor cells unhampered. Consequently, these CD8^+^ T cells produce IFN-γ and keep the tumor growth under control. Indeed, tumor growth is less pronounced in this situation (Figure 3B). Since production of IFN-γ leads to the expression of interferon-inducible immune suppressive factors such as PD-L1, we hypothesized that the shRNA targeting PD-L1 in tumor cells is not potent enough to mediate degradation of the newly transcribed mRNA. To evaluate this hypothesis, we treated PD-L1 knock-down TC-1 cells with 50 or 100 ng/ml IFN-γ *in vitro* and showed upregulation of PD-L1 in flow cytometry (Supplementary Figure 3A). We furthermore inoculated wild type mice that were depleted of CD8^+^ T cells with PD-L1 knock-down TC-1 cells. Tumor growth and PD-L1 expression on cancer and immune cells was evaluated. Tumor growth was enhanced in mice depleted of CD8^+^ T cells (Supplementary Figure 3B). Expression of PD-L1 was significantly lower on both immune cells and cancer cells in mice lacking CD8^+^ T cells (Supplementary Figure 3C), suggesting that indeed CD8^+^ T cells induce PD-L1 expression most likely through secretion of IFN-γ.

**Figure 4:**
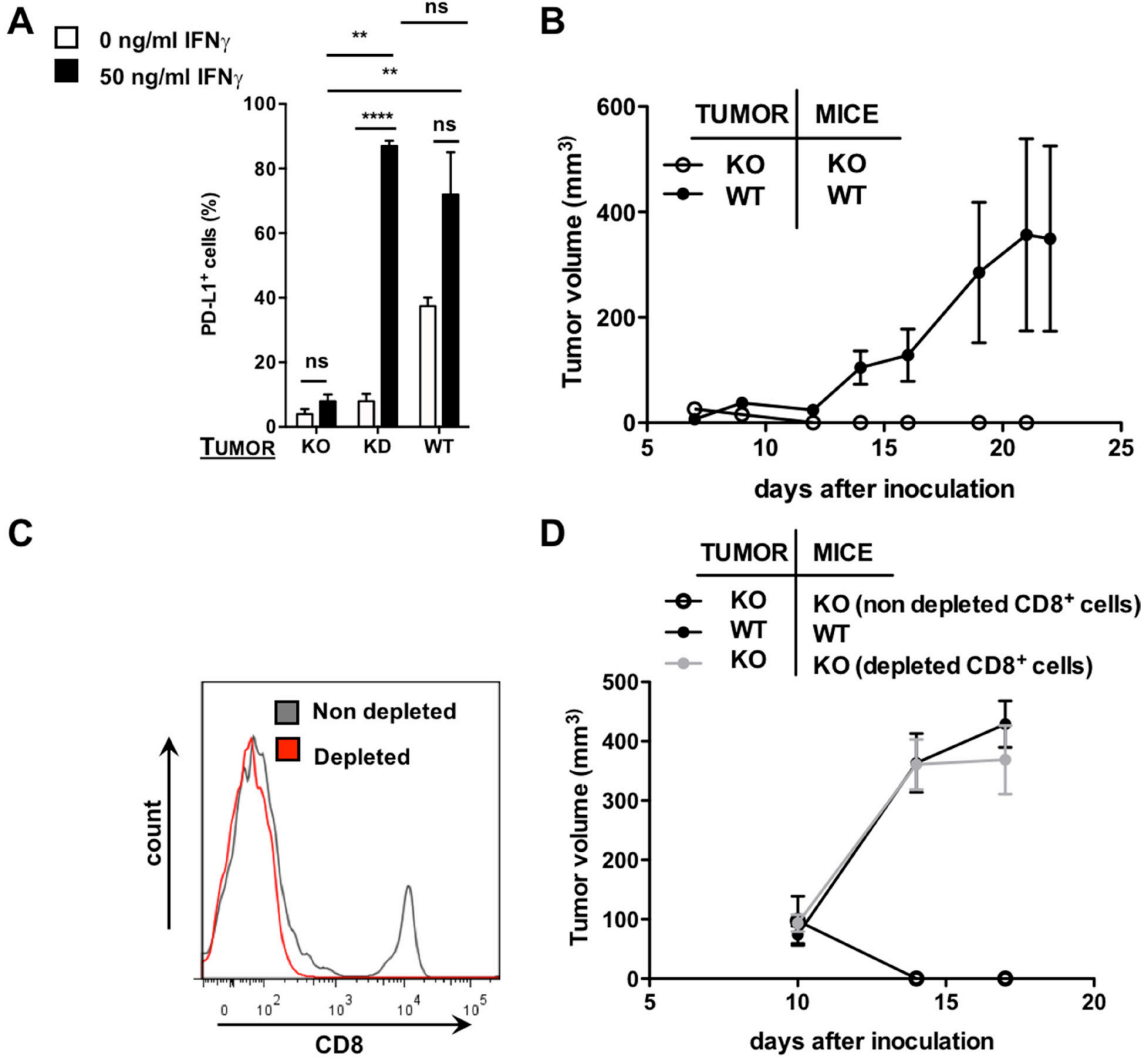
Use of the CRISPR/Cas9 technology to generate a PD-L1 KO tumor model (**A**) Percentage of PD-L1 as assessed with flow cytometry on TC-1 cells transduced with lentiviral vectors harboring CRISPR/Cas9 targeted to mouse PD-L1 (knock-out, KO) compared to WT and KD TC-1 cells either or not pre-treated with recombinant mouse IFN-γ (50 ng/mL) (*n* = 3). The graph summarizes the percentage PD-L1 positive cells as mean ± SEM. (**B**) TC-1 KO or WT cells were injected subcutaneously at the tail base of WT or PD-L1 KO mice. Tumor growth was followed every other day. The tumor size in function of time is shown as mean ± SEM (*n* = 3). (**C**) TC-1 KO cells were injected subcutaneously at the tail base of PD-L1 KO mice, which were pretreated with a CD8^+^ depleting antibody or an isotype matched control antibody. The percentage CD8 positive cells in the blood was evaluated using flow cytometry. The graph shows a representative histogram of CD8 positive cells in mice pretreated with an isotype matched control antibody (grey line) or a CD8 depleting antibody (red line). (**D**) TC-1 KO cells were injected subcutaneously at the tail base of PD-L1 KO mice, which were pretreated with a CD8^+^ depleting antibody or an isotype matched control antibody. WT TC-1 cells were injected subcutaneously at the tail base of WT mice. Tumor growth was followed every other day. The tumor size in function of time is shown as mean ± SEM (*n* = 3).

Despite the more complex interpretation of the data in Figure 3, these results do suggest that Nb C3 and E2 can be used to detect differences in PD-L1 expression in tumors. To further confirm the diagnostic potential of these Nbs in a more clear-cut PD-L1-negative tumor model, we decided to take an alternative approach and knocked out PD-L1 in TC-1 cells using the CRISPR/Cas9 technology. Knock-out of PD-L1 on these TC-1 cells, even in the presence of IFN-γ, was shown *in vitro* by flow cytometry, in contrast to TC-1 knock-down cells, which show upregulation of PD-L1 as a response to IFN-γ (Figure 4A). To generate a PD-L1 positive tumor model, we transplanted wild type TC-1 cells subcutaneously in wild type mice, while we transplanted PD-L1 knock-out TC-1 cells to PD-L1 knock-out or wild type mice to generate a PD-L1 negative tumor model. Initially tumor growth was observed in all conditions.

**Figure 5:**
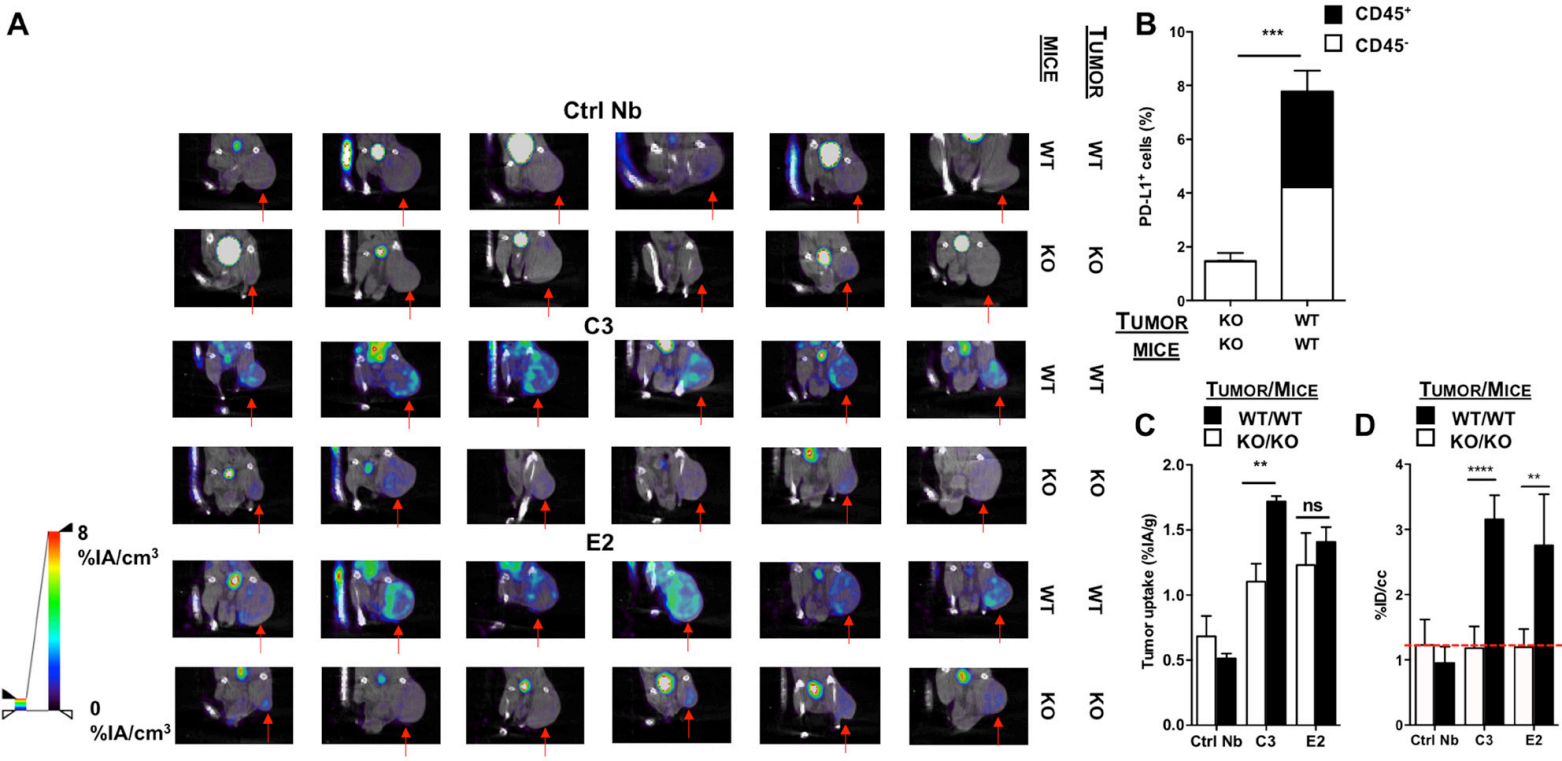
SPECT/CT in the CRISPR/Cas9-modified TC-1 model 1 hour after injection of ^99m^Tc-Nbs C3 or E2 (**A**) Images of the SPECT/CT scans to evaluate ^99m^ Tc-Nbs C3 and E2 for tumor stratification in CD8-depleted PD-L1 KO mice bearing PD-L1 KO tumors (KO) or WT mice bearing WT (PD-L1^+^) tumors (WT) (*n* = 6). The red arrow indicates the tumor on the images. (**B**) CD8-depleted PD-L1 KO mice injected with PD-L1 KO tumors or WT mice injected with WT (PD-L1^+^) TC-1 cells were sacrificed on day 17 and tumors were isolated, after which expression of PD-L1 on cancer cells (CD45^−^, white bar) and tumor-infiltrating immune cells (CD45^+^, black bar) was evaluated in flow cytometry. The graph summarizes the percentage of PD-L1 as mean ± SEM (*n* = 6). (**C**) Results of the gamma counting of isolated organs from CD8-depleted PD-L1 KO mice bearing PD-L1 KO tumors (KO, white bars) or WT mice bearing WT (PD-L1^+^) tumors (WT, black bars) injected with ^99Tm^ Tc-Nbs C3 and E2. The graph summarizes the %IA/g as mean ± SEM (*n* = 6). (**D**) Tumor uptake (%ID/cc) calculated via ROI analysis on the periphery of the tumor from CD8-depleted PD-L1 KO mice bearing PD-L1 KO tumors (KO, white bars) or WT mice bearing PD-L1^+^ tumors (WT, black bars) injected with ^99Tm^ Tc-Nbs C3 and E2. The graph summarizes the %ID/cc as mean ± SEM (*n* = 6).

However, from day 7 onwards tumors grown from the PD-L1 knock-out TC-1 cells in PD-L1 knock-out mice (Figure 4B) as well as in wild type mice (data not shown) regressed, again showing the central role of intratumoral PD-L1 expression in tumor development. To evaluate whether the tumor regression was due to an adaptive immune response, we performed an antibody-mediated depletion of CD8^+^ cells in PD-L1 knock-out mice prior to their inoculation with PD-L1 knock-out TC-1 cells. The depletion of CD8^+^ T cells was repeated at a three-day interval and was confirmed by flow cytometry (Figure 4C). Tumor growth was observed in these mice, while no tumor growth was observed in mice treated with isotype matched control antibodies (Figure 4D). We then compared by flow cytometry the expression of PD-L1 in these tumors to the expression of PD-L1 in tumors grown from TC-1 wild type cells in wild type mice. We showed that PD-L1 knock-out TC-1 tumors grown in CD8^+^-depleted PD-L1 knock-out mice had much lower PD-L1 expressing cells than wild type TC-1 tumors grown in wild type mice (Figure 5B), although the percentage PD-L1 positive cells in the latter tumors was rather low (similar to tumors in patients).

This model was used to perform SPECT/CT imaging 1 hour after injection of the ^99m^Tc-labeled Nbs C3 and E2, followed by evaluation of Nb accumulation in individual organs. Dissection and biodistribution data obtained at 80 minutes after Nb injection using γ-counting is shown in Supplementary Table 4 (on the right). Tissue-to-blood ratios of all organs, derived from the biodistribution data, are shown in Supplementary Table 5 (on the right). When analyzing the images, signals were especially observed at the periphery of the tumor in the PD-L1 positive tumor model. In contrast, the PD-L1 negative model showed lower signal in the tumor (Figure 5A). Remarkably, in the *ex vivo* analysis, significantly higher uptake of Nb C3, but not E2, in the TC-1 wild type tumor model compared to the TC-1 knock-out tumor model was detected (Figure 5C).

We then quantified ROI values at the periphery of the tumor to assess the accuracy of the ^99m^Tc-labeled Nbs to imagine PD-L1 expression levels in the tumor. These data revealed higher uptake of the Nb at the periphery of the tumor in TC-1 wild type tumors, where PD-L1 was expressed albeit at a rather low level, compared to the TC-1 knock-out tumors, where PD-L1 expression was almost negative (Figure 5D). These data confirmed the heterogeneity of PD-L1 expression in the tumor and showed that Nbs can be used to visualize PD-L1 in the tumor, even when PD-L1 expression is low, representing the clinical situation. These data confirm the importance to develop a non-invasive tool to evaluate PD-L1 expression in cancer patients before and during treatment.

## DISCUSSION

### Relevance of the study

Several immune checkpoint inhibitors targeting the PD-1/PD-L1 axis have been FDA approved because they have yielded unprecedented progression-free survival in a number of late stage cancer patients [7, 11, 20]. However, despite encouraging clinical results, a significant number of cancer patients do not respond to the therapy. It is believed that lack of PD-1/PD-L1 checkpoint expression is at least for a subset of these non-responders responsible for the therapy failure. Therefore, there is a compelling need to develop tools to visualize the PD-1/PD-L1 axis and predict which patients are likely to respond to PD-1/PD-L1 blocking immunotherapy.

Currently, IHC is applied to measure and evaluate PD-L1 expression [4, 38]. However, IHC has several limitations, as it is an invasive technique that does not allow visualizing the heterogeneous expression of PD-L1 in primary tumors. Furthermore, given the dynamic nature of PD-L1 expression during the disease course and in reaction to treatment, repeated biopsies with associated cumulative risks are necessary to fully capture the role of PD-L1 in predicting treatment response. Isotope-based imaging such as PET and SPECT is a non-invasive technique that allows repetitive image-based characterization of primary and metastatic lesions independent of their location. This has been shown for multiple cancer cell associated antigens, that are typically abundantly available on the tumor cell membrane [39–43]. The challenge is however much greater if the antigen is present at lower levels and only on a subset of tumor cells. It remains to be proven that also there, nuclear medicine can accurately assess antigen expression levels.

### Rationale to use Nbs for the imaging of immune checkpoints

We report on the generation and selection of Nbs for quantitative, non-invasive *in vivo* imaging of the inhibitory ligand PD-L1. Compared to monoclonal antibodies, Nbs show superior imaging characteristics, with fast blood clearance resulting in high contrast images within only hours after injection of the tracer, as was recently shown in a clinical trial using anti-HER2 Nbs for PET/CT imaging [44]. When full monoclonal antibodies are used for imaging, imaging is typically performed after 4 to 6 days, resulting in the use of long-lived isotopes and increased radiation exposure to the subject [45]. Even at these time points, remaining non-specific signal due to uncleared tracer in the blood could result in insufficient accuracy to correctly assess low-expressed targets such as PD-L1. We are therefore convinced that anti-PD-L1 Nbs offer important advantages compared to radiolabeled antibodies for imaged-based characterization of lesions.

Nbs C3 and E2 were selected as lead compounds based on their antigen-specificity, nanomolar affinity and distribution in healthy mice, showing accumulation in the lungs, heart, thymus, spleen, lymph nodes and brown adipose tissue, next to kidneys, bladder and liver.

The signals in kidneys and urinary bladder are observed in all mice and is a typical feature of Nbs, as they are cleared by renal uptake and elimination due to their small size [46]. The signals in liver are partially specific and partially aspecific. We observed that the liver retention differs between the C- and E-family with higher signals observed for Nb C3 than for Nb E2. This can be explained by the Nb composition, which determines its metabolism and as such the degree of liver uptake. The specific signals, showing distribution of the Nbs in lungs, heart, thymus, spleen, lymph nodes and brown adipose tissue, was previously described using mAbs as a tracer [31]. This distribution is linked to the role of the PD-1/PD-L1 immune checkpoint, which under healthy conditions is at work during the induction of peripheral T cell self-tolerance and in limiting effector T cell induction [47, 48]. The uptake in brown adipose tissue has only recently been observed, and was confirmed by our imaging experiments [31].

### Development of models to evaluate Nb tracers with high potential for imaging

To study the Nbs as tracers to detect PD-L1 expression in tumors, we set out to generate *in vivo* tumor models with low and high PD-L1 expression. We used the tumor cell line TC-1, a lung epithelial cell line, because we previously showed that TC-1 cells expresses PD-L1 and exploit this mechanism to counteract effector T cells that express high levels of PD-1 [49]. Moreover, in the clinic it was shown in lung cancer that responses to anti-PD-L1 and PD-1 treatment correlate with PD-L1 expression in the tumor [4]. First, we used the RNAi technology to knock-down PD-L1 expression. Growing these cells in immunocompetent wild type mice confirmed the critical role of PD-L1, as the shRNA-modified TC-1 cells progressed slower than the PD-L1 overexpressing TC-1 cells. Against our expectations, we observed higher accumulation of the Nbs in tumors grown from shRNA-modified TC-1 cells. Using flow cytometry, we confirmed that a higher percentage of immune and tumor cells expressed PD-L1. We hypothesize that the unexpected high expression of PD-L1 in this model can be explained as follows: initially shRNA-modified TC-1 cells show low PD-L1 expression, and as such are infiltrated with CD8^+^ T cells that are unhampered in their ability to produce IFN-γ. As IFN-γ triggers the transcription of PD-L1 [25], the tumor cells will produce an abundant amount of PD-L1 mRNA. Most likely, the shRNA is not efficient enough to tackle the abundance of PD-L1 mRNA. Consequently, the tumor cells become PD-L1 positive. It is indeed described that PD-L1 is up-regulated to mediate resistance to an adaptive immune response [50]. We were able to show the important role of tumor-infiltrating lymphocytes and their IFN-γ secretion in the local PD-L1 expression on tumor cells. Therefore, we inoculated wild type mice that were depleted of CD8^+^ T cells with PD-L1 knock-down tumor cells. We observed that the expression of PD-L1 was significantly lower on both immune cells and cancer cells in mice lacking CD8^+^ T cells, suggesting that indeed CD8^+^ T cells induce PD-L1 expression most likely through secretion of IFN-γ.

We then altered our strategy and used the CRISPR/Cas9 technology to knock out PD-L1. *In vitro*, we showed that even in the presence of IFN-γ, PD-L1 expression was low. When growing these tumor cells in PD-L1 knock-out mice, we observed palpable tumors at day 7, which regressed later on. This was unexpected as the CRISPR/Cas9 technology has been previously used to generate PD-L1 knock-out tumor cells or PD-1 negative T cells without hampering tumor growth or viability [31, 51]. This observation again highlights the critical role of PD-L1 in the development of the TC-1 lung epithelial tumors. We hypothesized that tumors lacking PD-L1 were most likely rejected by infiltrating CD8^+^ T cells that are unhampered in their activity. Therefore, we depleted the CD8^+^ T cells using a CD8 depleting antibody. We observed tumor growth, thereby validating our hypothesis. Moreover, we showed that these tumors were void of PD-L1 expressing cells.

When evaluating the ^99m^Tc-labeled lead Nbs as a tracer in TC-1 tumors with varying PD-L1 expression, we observed that the strength of the signal on the SPECT/CT images correlated well with the levels of PD-L1 expression *ex vivo* evaluated using flow cytometry, for the knock-down tumor model. This model had relatively high level of PD-L1 expressing cells in the tumor, typically between 30 and 50%.

Using the knock-out tumor model, the level of PD-L1 expressing cells was much lower, on average around 8% of cells in the positive tumor and below 2% in the negative tumor model. However, even in this low-expressing model, this small difference in expression levels was visually discernable as a high uptake at the periphery of the tumor and confirmed by image quantification. Using *ex vivo* quantification of the radioactive content of the entire tumor, the difference was only retained for Nb C3, but not for E2, probably because the uptake is averaged out over the entire tumor rather than looking at the focal expression. Given the better results of C3 compared to E2 in this low level expression model, C3 is selected as the lead compound with the highest potential to correctly assess clinically relevant differences in PD-L1 expression levels. As such, these Nbs with a high affinity for mouse PD-L1 are suited to evaluate the critical role of PD-L1 in immunocompetent mice bearing syngeneic tumor models. Unfortunately, the selected Nbs do not show sufficient cross-reactive binding to human PD-L1 for clinical translation. Therefore, there is a need to validate Nbs that bind with high affinity to human PD-L1 as a diagnostic tool for the selection of patients.

Other research teams have developed imaging strategies for PD-1 or PD-L1 in the recent past. Multiple have investigated mAbs as imaging agents, with disadvantages associated with the slow blood clearance of such tracers [26–31]. A recent study used high-affinity consensus (HAC)-PD-1 radiotracer variants to image PD-L1 expression in the tumor as early as one hour after injection. These radiotracers, with a size of 14 kDa and a dissociation constant of 100 pM, were tested in a human CT26 PD-L1 negative tumor model and tumor model engineered to constitutively express human PD-L1. These tracers were the first engineered binders to distinguish between PD-L1 positive and negative tumors. The properties of these radiotracers are close to the properties of Nbs, as Nbs have a size of 15 kDa and bind with nanomolar affinity to their target. The here-presented Nbs are less cross-reactive to human PD-L1 than some of the HAC-PD-1 tracer variants. The HAC-PD-1 variants showed non-specific background signal in liver and spleen, which we also observed with our Nbs. However, these tracers showed a lower tumor-to-blood ratio compared to our Nbs. Furthermore, the difference in uptake between the negative and positive PD-L1 tumor is rather lower for the HAC-PD-1 variant [33].

In conclusion, we show that Nbs can be used to non-invasively and quantitatively image PD-L1 in the tumor as soon as 1 hour after its injection. In addition, they can be used to evaluate the critical role of PD-L1 in syngeneic tumor models, resembling the patient's situation. Moreover, this study provides a rationale to further develop Nbs that bind human PD-L1 as a diagnostic tool for patient selection and potentially treatment monitoring, as Nb radiotracers exhibit many favourable imaging characteristics.

## MATERIALS AND METHODS

### Reagents

Anti-HA mAb (Biolegend, clone 16B12) was used in ELISA and in flow cytometry to detect HA-tagged Nbs in periplasmic extracts (PEs); anti-His mAb (AbD Serotec, clone AD1.1.10), followed by a phycoerythrin labeled anti-mouse IgG antibody (BD biosciences, clone A85-1), was used in flow cytometry to detect binding of purified His-tagged Nbs. All Biacore consumables were from GE Healtcare. Recombinant mouse PD-L1 protein (Sino Biological #50010-M08H) was used for biopanning and initial enzyme-linked immunosorbant assay (ELISA) screening. Mouse or human PD-L1 Fc-fusion proteins (R&Dsystems, 1019-B7 and 156- B7 respectively) were used for calibration-free concentration analysis (CFCA) and affinity estimations of the Nbs in PEs. Mouse or human PD-L1 His-tagged proteins (Sino Biological #50010-M08H and #10084-H08H) were used for ELISA screenings of Nbs in PEs and Surface Plasmon Resonance (SPR) affinity determinations on purified Nbs. A phycoerythrin labeled antibody specific for mouse PD-L1 (Biolegend, 10F.9G2) or an allophycocyanin labeled antibody specific for human PD-L1 (eBioscience, MIH5) was used in flow cytometry to evaluate PD-L1 expression on cells *in vitro*. Furthermore, an HorizonV450 labeled antibody specific for mouse CD45 (Biologend, 30-F11) and a phycoerythrin labeled antibody specific for mouse PD-L1 (Biolegend, 10F.9G2) was used to evaluate PD-L1 expression in flow cytometry.

### Isolation of PD-L1-specific Nbs

A dromedary was subcutaneously immunized 5 times bi-weekly, each time with 10 million RAW264.7 cells. Peripheral blood lymphocytes were purified and used as a source to create a Nb-phage display library as described previously [52]. Mouse PD-L1 reactive Nbs were identified by biopanning of this library and ELISA screenings of PEs of individual Nb clones on recombinant mouse PD-L1 protein, and sequence analysis following published protocols [52].

### Periplasmic extractions of small-scale cultures

PEs were produced as described [53]. Briefly, E.coli WK6 cells were transformed with Nb-coding pHEN4 plasmids and cultured in 10 mL Terrific broth (TB) containing 100 μg/mL Ampicillin (Fermentas) for 6h at 37°C. Nb expression was induced overnight with 1 mM isopropyl-β-D-thiogalactopyranoside (IPTG) while shaking at 200 rpm. Cultures were centrifuged for 10 min at 1643xg and 4°C, after which the bacterial pellet was frozen at −80°C. One day later, 2 mL phosphate buffered saline (PBS, Sigma-Aldrich) was added to the thawed bacterial pellets and pellets were stirred for 30 min at 4°C. The released periplasmic proteins were collected by centrifugation for 20 minutes at 730 × g and 4°C, followed by 0.22 μm filtration (Millipore).

These PEs were used to evaluate interaction with mouse and human PD-L1 (*i*) in ELISA to assess qualitative binding to immobilised recombinant PD-L1 proteins; (*ii*) using SPR to rank Nbs for binding to recombinant PD-L1 proteins; (*iii*) in flow cytometry to assess binding to HEK293T cells transfected with PD-L1 cDNAs. ELISA on PEs are performed as described elsewhere [52]. SPR and flow cytometry analyses of PEs are described below.

### Large-scale production, purification and quality control of Nbs

cDNAs of selected Nbs were recloned into the vector pHEN6 to encode a C-terminal His_6_ tag. Also Nb BCII10 and R3B23, specific for bacterial β-lactamase and 5T2 multiple myeloma paraprotein respectively, were produced and used throughout the study as negative controls [54] [55]. All Nbs were produced and purified in PBS as described previously [52]. Protein purity was assayed by 12% sodium dodecyl sulfate-polyacrylamide gel electrophoresis (SDS-PAGE) under reducing conditions, followed by staining with Coomassie Blue. Endotoxins were measured in the LAL (Limulus amebocyte lysate assay). The latter was performed as recommended by the manufacturer (Pierce^TM^ LAL Chromogenic Endotoxin Quantification Kit, Thermofisher).

### Surface plasmon resonance

All measurements were performed on a Biacore T200 device (GE Healtcare) at 25°C and using Hepes-buffered saline (HBS; 0.01 M HEPES pH 7.4, 0.15 M NaCl, 3 mM EDTA, 0.005% Tween 20) as running buffer. All recombinant proteins were dissolved to 10 ug/mL in 10mM Na-acetate pH 5.0 for immobilization on a CM5 sensor chip using linkage chemistry with 1-(3-(dimethylamino)propyl)-3-ethylcarbodiimide (EDC) and N-hydroxy-succinimide (NHS). Unreacted EDC-NHS linkers were blocked with 1M ethanolamine-HCl. For all measurements, SPR signals in the flow cell with immobilized protein were subtracted with those in a flow cell that underwent the same manipulations but where recombinant protein was omitted, to obtain specific binding signals (response units, RU). For CFCA, recombinant mouse PD-L1 protein was coupled to 9920 RU, for all other measurements between 650 and 900 RU proteins were coupled. For all measurements and between each cycle, chips were regenerated twice with 0.5M NaCl, 15 mM NaOH, each time for 10 sec at 40 μl/min. CFCA was used to estimate the concentration of Nb in each PE and was performed according to manufacturer's instructions. Briefly, 3 different dilutions of PEs (undiluted, 10 and 100-fold diluted in HBS) were run for 35 sec at 5 and at 100 μL/min on a chip with high mouse PD-L1 protein densities and RUs were recorded. The Nbs' diffusion coefficient was calculated using an online tool provided by the company, assuming a protein globular shape, and ranged between 1.0 and 1.3E^−10^ m^2^/s depending on the theoretical Nb molecular weight. The PE dilution resulting in the largest difference in slope between the two flow speeds was used to calculate the Nb concentration using Biacore Evaluation software.

The estimated Nb concentrations in the PEs ranged between 13 and 4900 nM and was usually around 150 nM. Nbs, either in PEs or as purified proteins, were tested for affinity on immobilized mouse or human PD-L1 protein in SPR. To this end, 9 different Nb dilutions were allowed to bind to the target protein for 120 sec and dissociation was monitored for 160 sec. The equilibrium dissociation constant K_D_ was calculated by fitting the obtained sensorgrams to theoretical curves, assuming 1-to-1 binding geometries, using Biacore Evaluation software.

### Mice and cell lines

Mice deficient in PD-L1 expression (referred to as PD-L1 knock-out mice) were bred in-house, C57BL/6 mice were supplied by Charles River Laboratories (France) at 6 weeks of age. All experiments were performed in accordance to the European guidelines for animal experimentation under a License LA1230214. Experiments were approved by the Ethical Committee for the use of laboratory animals of the Vrije Universiteit Brussel (15-214-1).

T.C. Wu (Johns Hopkins University, Baltimore, Maryland, United States of America) kindly provided the mouse lung epithelial cell line TC-1. TC-1 cells were cultured in RPMI medium, consisting of RPMI 1640 medium (Sigma-Aldrich) supplemented with 10% FCI serum (Harlan), 2 mmol/L L-glutamine (L-Glu; Sigma-Aldrich), 100 U/mL penicillin, 100 μg/mL streptomycin (PS; Sigma-Aldrich), 1 mmol/L sodium pyruvate and non-essential amino acids (Sigma-Aldrich). Lentiviral vectors harboring the mouse PD-L1 gene, a short hairpin RNA (shRNA) targeting PD-L1 or a PD-L1 targeting guide RNA and Cas9 were used to generate TC-1 lung epithelial cells in which PD-L1 was knocked-in, knocked-down or knocked-out respectively. Lentivirus generation is described below.

Human embryonal kidney (HEK) 293T cells were purchased from the American Type Culture Collection (ATCC/LGC standards) and were cultured in Dulbecco's modified Eagle's medium (Sigma-Aldrich) supplemented with 10% FBS (Harlan), L-Glu and PS. Lentiviral vectors harboring the mouse or human PD-L1 gene were used to generate HEK293T cells expressing mouse or human PD-L1.

B16 cells were purchased from ATCC and cultured in DMEM medium, consisting of Dulbecco's modified Eagle's medium (Sigma-Aldrich) supplemented with 10% FBS (Harlan), L-Glu, PS and 50 μM β-mercaptoethanol. Lentiviral vectors harboring shRNA targeting PD-L1 were used to generate a B16 cell line negative for mouse PD-L1 (PD-L1-). Parental B16 cells served as a mouse PD-L1 positive cell line (PD-L1+).

### Lentiviral vector production, characterization and transduction

The plasmids pCMVΔR8.9 and pMD.G were a gift from D. Trono (Ecole Polytechnique Fédéral de Lausanne, Swiss). The transfer plasmid encoding human PD-L1, pHR’-huPD-L1 was previously described [23]. The plasmid encoding mouse PD-L1, pHR’-moPD-L1, was generated similarly as pHR’-huPD-L1. The transfer plasmid harboring shRNA against mouse PD-L1 was previously described [56]. The mouse CD274 sgRNA CRISPR/Cas9 'All-in-One' lentiviral transfer vector was purchased from Applied Biological Materials Inc. The production of lentiviral vectors and their characterization by flow cytometry was performed as described [57]. Transduction of HEK293T, TC-1 and B16 cells was carried out at a multiplicity of infection (MOI) of 10, using the protocol described to transduce human dendritic cells [58]. Non-transduced cells are referred to as wild type cells. TC-1 cells transduced with lentiviral vectors harboring mouse PD-L1 are referred to as TC-1 PD-L1 knock-in cells. TC-1 cells transduced with a lentiviral vector harboring shRNA against mouse PD-L1 are referred to as TC-1 PD-L1 knock-down cells and TC-1 cells transduced with lentiviral vectors harboring CRISPR/Cas9 targeted to mouse PD-L1 are referred to as TC-1 PD-L1 knock-out cells.

### Flow cytometry

Expression of PD-L1 on wild type or lentivirally modified HEK293T, TC-1 or B16 cells was evaluated using an antibody specific for mouse or human PD-L1. Binding of purified His-tagged Nbs or HA-tagged Nbs in PEs to wild type or lentivirally modified HEK293T cells was detected with respectively an anti-His-tag antibody or an anti-HA-tag antibody followed by an anti-mouse IgG antibody. Single cell-suspensions of spleens, lymph nodes, tumors and brown adipose tissue were prepared and expression of PD-L1 on immune cells versus non-immune cells was evaluated using an antibody specific for mouse CD45 and an antibody specific for mouse PD-L1. Cells were acquired on the LSRFortessa flow cytometer (BD Biosciences) and data were analyzed with FACSDiva (BD Biosciences) or FlowJo (Tristar Inc.) software.

### Tumor challenge

C57BL/6 wild type and PD-L1 knock-out mice were injected subcutaneously with 5 × 10^5^ TC-1 wild type, PD-L1 knock-in, knock-down or knock-out cells. When indicated, mice were injected one day before tumor inoculation with 100 μg depleting anti-CD8 antibodies (clone 2.43) or isotype matched control antibodies (LTF-2). The injections were repeated every three days. The antibodies were purchased from BioXcell. The tumor volume was measured thrice a week using an electronic caliper. The tumor volume was calculated using the following formula: (length × width^2^)/2.

### ^99m^Tc-Nb labeling, pinhole SPECT-micro-CT imaging and image analysis

The Nbs were labeled as described by Xavier et al [40]. Briefly, the Nbs were coupled to the C-terminal His_6_-tag with ^99m^Tc-tricarbonyl intermediate [^99m^Tc(H_2_O)_3_(CO)_3_]^99m^, which was synthesized using the Isolink® labelling kit (Mallinckrodt Medical BV). The ^99m^Tc-Nb solution was purified on a NAP-5 column (GE Healthcare) pre-equilibrated with PBS to remove unbound (^99m^Tc(H_2_0)_3_(CO)_3_)^+^ and finally filtered through a 0.22 μm filter (Millipore) to remove aggregates. The labeling efficiency was determined both directly after labeling and after purifications by instant thin-layer chromatography (iTLC) with 100% acetone as the mobile phase. Mice were injected intravenously with 100 to 200 μL of 45 to 155 MBq of ^99m^Tc-labeled Nbs (10 μg), one hour prior to pinhole SPECT-micro-CT imaging. Imaging was performed as described [59]. Total body pinhole SPECT was performed once using a dual-head γ-camera (e.cam180; Siemens Medical Solutions), mounted with 2 multipinhole collimators (three 1.5 mm pinholes in each collimator, 200 mm focal length, and 80 mm radius of rotation). Micro-CT was performed using a dual-source CT scanner (Skyscan 1178; Skyscan) with 60 kV and 615 mA at a resolution of 83 μm. CT images were reconstructed using filtered back projection (NRecon; Skyscan). SPECT images were reconstructed using an iterative reconstruction algorithm (ordered-subset expectation maximization) modified for the 3-pinhole geometry and automatically reoriented for fusion with CT images based on six ^57^Co landmarks [60]. Images were further visually analyzed and quantified where appropriate using AMIDE (Medical Image Data Examiner software) [61]. Quantification of tracer uptake in the center and at the periphery of tumors was performed using 4 cubic 3 × 3 × 3 mm regions of Interest (ROIs) positioned on the 3 areas with highest uptake in the periphery and 1 ROI positioned on area with the lowest uptake in the center of the tumor. For the periphery, the average of the 3 ROI's was calculated and represents the uptake in the tumor periphery. Twenty minutes after imaging, mice were sacrificed and all organs (including tumors when applicable) were isolated to measure radioactivity using a γ-counter (Cobra Inspector 5003, Canberra, Packard). The amount of radioactivity in organs is expressed as percent injected activity per gram (%IA/g).

### Statistics

Results are expressed as mean ± standard error of the mean. A non-parametric Mann-Whitney U test or One-Way-ANOVA followed by Bonferroni multicomparison test was carried out to compare data sets. Sample sizes and number of times experiments were repeated are indicated in the figure legends. The number of asterisks in the figures indicates the statistical significance as follows: *P < 0.05; **P < 0.01; ***P < 0.001.

## SUPPLEMENTARY MATERIALS FIGURES AND TABLES









## Abbreviations

Nbs: Nanobodies
^99m^Tc: Technetium-99m
PD-1: programmed death-1
PD-L1: programmed death-ligand 1
mAbs: monoclonal antibodies
IHC: immunohistochemistry
HAC: high-affinity consensus
PEs: periplasmatic extracts
ELISA: enzyme-linked immunosorbant assay
CFCA: calibration-free concentration analysis
SPR: Surface Plasmon Resonance
TB: Terrific broth
IPTG: isopropyl-β-D-thiogalactopyranoside
PBS: phosphate buffered saline
SDS: sodium dodecyl sulfate-polyacrylamide gel electrophoresis
LAL: Limulus amebocyte lysate assay
PS: Penicillin/Streptomycin
shRNA: short hairpin RNA
HEK: Human embryonal kidney
ATCC: American Type Culture Collection
MOI: multiplicity of infection
iTLC: instant thin-layer chromatography

## References

[1] Coulie PG, Van den Eynde BJ, van der Bruggen P, Boon T (2014). Tumour antigens recognized by T lymphocytes: at the core of cancer immunotherapy. Nat Rev Cancer.

[2] Pen JJ, Aerts JL, Liechtenstein T, Escors D, Breckpot K (2014). Manipulating Immune Regulatory Pathways to Enhance T Cell Stimulation. Immune Response Activation.

[3] Brahmer JR, Drake CG, Wollner I, Powderly JD, Picus J, Sharfman WH, Stankevich E, Pons A, Salay TM, McMiller TL, Gilson MM, Wang C, Selby M (2010). Phase I study of single-agent anti-programmed death-1 (MDX-1106) in refractory solid tumors: safety, clinical activity, pharmacodynamics, and immunologic correlates. J Clin Oncol.

[4] Topalian SL, Hodi FS, Brahmer JR, Gettinger SN, Smith DC, McDermott DF, Powderly JD, Carvajal RD, Sosman JA, Atkins MB, Leming PD, Spigel DR, Antonia SJ (2012). Safety, activity, and immune correlates of anti-PD-1 antibody in cancer. N Engl J Med.

[5] Wolchok JD, Kluger H, Callahan MK, Postow MA, Rizvi NA, Lesokhin AM, Segal NH, Ariyan CE, Gordon RA, Reed K, Burke MM, Caldwell A, Kronenberg SA (2013). Nivolumab plus ipilimumab in advanced melanoma. N Engl J Med.

[6] Ansell SM, Lesokhin AM, Borrello I, Halwani A, Scott EC, Gutierrez M, Schuster SJ, Millenson MM, Cattry D, Freeman GJ, Rodig SJ, Chapuy B, Ligon AH (2015). PD-1 blockade with nivolumab in relapsed or refractory Hodgkin's lymphoma. N Engl J Med.

[7] Weber JS, D’Angelo SP, Minor D, Hodi FS, Gutzmer R, Neyns B, Hoeller C, Khushalani NI, Miller WH, Lao CD, Linette GP, Thomas L, Lorigan P (2015). Nivolumab versus chemotherapy in patients with advanced melanoma who progressed after anti-CTLA-4 treatment (CheckMate 037): a randomised, controlled, open-label, phase 3 trial. Lancet Oncol.

[8] Gettinger SN, Horn L, Gandhi L, Spigel DR, Antonia SJ, Rizvi NA, Powderly JD, Heist RS, Carvajal RD, Jackman DM, Sequist LV, Smith DC, Leming P (2015). Overall Survival and Long-Term Safety of Nivolumab (Anti-Programmed Death 1 Antibody, BMS-936558, ONO-4538) in Patients With Previously Treated Advanced Non-Small-Cell Lung Cancer. J Clin Oncol.

[9] Brahmer J, Reckamp KL, Baas P, Crinò L, Eberhardt WE, Poddubskaya E, Antonia S, Pluzanski A, Vokes EE, Holgado E, Waterhouse D, Ready N, Gainor J (2015). Nivolumab versus Docetaxel in Advanced Squamous-Cell Non-Small-Cell Lung Cancer. N Engl J Med.

[10] Hamid O, Robert C, Daud A, Hodi FS, Hwu WJ, Kefford R, Wolchok JD, Hersey P, Joseph RW, Weber JS, Dronca R, Gangadhar TC, Patnaik A (2013). Safety and tumor responses with lambrolizumab (anti-PD-1) in melanoma. N Engl J Med.

[11] Robert C, Schachter J, Long GV, Arance A, Grob JJ, Mortier L, Daud A, Carlino MS, McNeil C, Lotem M, Larkin J, Lorigan P, Neyns B, KEYNOTE-006 investigators (2015). Pembrolizumab versus Ipilimumab in Advanced Melanoma. N Engl J Med.

[12] Garon EB, Rizvi NA, Hui R, Leighl N, Balmanoukian AS, Eder JP, Patnaik A, Aggarwal C, Gubens M, Horn L, Carcereny E, Ahn MJ, Felip E, KEYNOTE-001 Investigators (2015). Pembrolizumab for the treatment of non-small-cell lung cancer. N Engl J Med.

[13] Armand P, Nagler A, Weller EA, Devine SM, Avigan DE, Chen YB, Kaminski MS, Holland HK, Winter JN, Mason JR, Fay JW, Rizzieri DA, Hosing CM (2013). Disabling immune tolerance by programmed death-1 blockade with pidilizumab after autologous hematopoietic stem-cell transplantation for diffuse large B-cell lymphoma: results of an international phase II trial. J Clin Oncol.

[14] Westin JR, Chu F, Zhang M, Fayad LE, Kwak LW, Fowler N, Romaguera J, Hagemeister F, Fanale M, Samaniego F, Feng L, Baladandayuthapani V, Wang Z (2014). Safety and activity of PD1 blockade by pidilizumab in combination with rituximab in patients with relapsed follicular lymphoma: a single group, open-label, phase 2 trial. Lancet Oncol.

[15] Powles T, Eder JP, Fine GD, Braiteh FS, Loriot Y, Cruz C, Bellmunt J, Burris HA, Petrylak DP, Teng SL, Shen X, Boyd Z, Hegde PS (2014). MPDL3280A (anti-PD-L1) treatment leads to clinical activity in metastatic bladder cancer. Nature.

[16] Herbst RS, Soria JC, Kowanetz M, Fine GD, Hamid O, Gordon MS, Sosman JA, McDermott DF, Powderly JD, Gettinger SN, Kohrt HE, Horn L, Lawrence DP (2014). Predictive correlates of response to the anti-PD-L1 antibody MPDL3280A in cancer patients. Nature.

[17] Larkin J, Chiarion-Sileni V, Gonzalez R, Grob JJ, Cowey CL, Lao CD, Schadendorf D, Dummer R, Smylie M, Rutkowski P, Ferrucci PF, Hill A, Wagstaff J (2015). Combined Nivolumab and Ipilimumab or Monotherapy in Untreated Melanoma. N Engl J Med.

[18] Brahmer JR, Tykodi SS, Chow LQ, Hwu WJ, Topalian SL, Hwu P, Drake CG, Camacho LH, Kauh J, Odunsi K, Pitot HC, Hamid O, Bhatia S (2012). Safety and activity of anti-PD-L1 antibody in patients with advanced cancer. N Engl J Med.

[19] Motz GT, Coukos G (2013). Deciphering and reversing tumor immune suppression. Immunity.

[20] Rexer H, Retz M, Albers P (2016). Adjuvante Studie beim muskelinvasiven Hoch-Risiko-Urothelkarzinom : Eine offene, multizentrische, randomisierte Phase-III-Studie mit Atezolizumab (Anti-PD-L1-Antikörper) als adjuvante Therapie im Vergleich mit einer Beobachtung bei Patienten mit muskelinvasivem Hoch-Risiko-Urothelkarzinom nach operativer Entfernung (IMvigor010) – Studie AB 53/15 der AUO. Urologe A.

[21] Wang X, Teng F, Kong L, Yu J (2016). PD-L1 expression in human cancers and its association with clinical outcomes. Onco Targets Ther.

[22] Teng MW, Ngiow SF, Ribas A, Smyth MJ (2015). Classifying Cancers Based on T-cell Infiltration and PD-L1. Cancer Res.

[23] Pen JJ, Keersmaecker BD, Heirman C, Corthals J, Liechtenstein T, Escors D, Thielemans K, Breckpot K (2014). Interference with PD-L1/PD-1 co-stimulation during antigen presentation enhances the multifunctionality of antigen-specific T cells. Gene Ther.

[24] Karwacz K, Bricogne C, MacDonald D, Arce F, Bennett CL, Collins M, Escors D (2011). PD-L1 co-stimulation contributes to ligand-induced T cell receptor down-modulation on CD8+ T cells. EMBO Mol Med.

[25] Ritprajak P, Azuma M (2015). Intrinsic and extrinsic control of expression of the immunoregulatory molecule PD-L1 in epithelial cells and squamous cell carcinoma. Oral Oncol.

[26] Heskamp S, Hobo W, Molkenboer-Kuenen JD, Olive D, Oyen WJ, Dolstra H, Boerman OC (2015). Noninvasive Imaging of Tumor PD-L1 Expression Using Radiolabeled Anti-PD-L1 Antibodies. Cancer Res.

[27] Maute RL, Gordon SR, Mayer AT, McCracken MN, Natarajan A, Ring NG, Kimura R, Tsai JM, Manglik A, Kruse AC, Gambhir SS, Weissman IL, Ring AM (2015). Engineering high-affinity PD-1 variants for optimized immunotherapy and immuno-PET imaging. Proc Natl Acad Sci USA.

[28] Josefsson A, Nedrow JR, Park S, Banerjee SR, Rittenbach A, Jammes F, Tsui B, Sgouros G (2016). Imaging, Biodistribution, and Dosimetry of Radionuclide-Labeled PD-L1 Antibody in an Immunocompetent Mouse Model of Breast Cancer. Cancer Res.

[29] Chatterjee S, Lesniak WG, Gabrielson M, Lisok A, Wharram B, Sysa-Shah P, Azad BB, Pomper MG, Nimmagadda S (2016). A humanized antibody for imaging immune checkpoint ligand PD-L1 expression in tumors. Oncotarget.

[30] Natarajan A, Mayer AT, Xu L, Reeves RE, Gano J, Gambhir SS (2015). Novel Radiotracer for ImmunoPET Imaging of PD-1 Checkpoint Expression on Tumor Infiltrating Lymphocytes. Bioconjug Chem.

[31] Hettich M, Braun F, Bartholomä MD, Schirmbeck R, Niedermann G (2016). High-Resolution PET Imaging with Therapeutic Antibody-based PD-1/PD-L1 Checkpoint Tracers. Theranostics.

[32] De Vos J, Devoogdt N, Lahoutte T, Muyldermans S (2013). Camelid single-domain antibody-fragment engineering for (pre)clinical in vivo molecular imaging applications: adjusting the bullet to its target. Expert Opin Biol Ther.

[33] Mayer AT, Natarajan A, Gordon S, Maute R, McCracken M, Ring A, Weissman I, Gambhir SS (2017). Practical ImmunoPET radiotracer design considerations for human immune checkpoint imaging. J Nucl Med.

[34] Muyldermans S (2013). Nanobodies: natural single-domain antibodies. Annu Rev Biochem.

[35] Ishida M, Iwai Y, Tanaka Y, Okazaki T, Freeman GJ, Minato N, Honjo T (2002). Differential expression of PD-L1 and PD-L2, ligands for an inhibitory receptor PD-1, in the cells of lymphohematopoietic tissues. Immunol Lett.

[36] Liang SC, Latchman YE, Buhlmann JE, Tomczak MF, Horwitz BH, Freeman GJ, Sharpe AH (2003). Regulation of PD-1, PD-L1, and PD-L2 expression during normal and autoimmune responses. Eur J Immunol.

[37] Rodig N, Ryan T, Allen JA, Pang H, Grabie N, Chernova T, Greenfield EA, Liang SC, Sharpe AH, Lichtman AH, Freeman GJ (2003). Endothelial expression of PD-L1 and PD-L2 down-regulates CD8+ T cell activation and cytolysis. Eur J Immunol.

[38] Taube JM, Anders RA, Young GD, Xu H, Sharma R, McMiller TL, Chen S, Klein AP, Pardoll DM, Topalian SL, Chen L (2012). Colocalization of inflammatory response with B7-h1 expression in human melanocytic lesions supports an adaptive resistance mechanism of immune escape. Sci Transl Med.

[39] Evazalipour M, D’Huyvetter M, Tehrani BS, Abolhassani M, Omidfar K, Abdoli S, Arezumand R, Morovvati H, Lahoutte T, Muyldermans S, Devoogdt N (2014). Generation and characterization of nanobodies targeting PSMA for molecular imaging of prostate cancer. Contrast Media Mol Imaging.

[40] Xavier C, Devoogdt N, Hernot S, Vaneycken I, D’Huyvetter M, De Vos J, Massa S, Lahoutte T, Caveliers V (2012). Site-Specific Labeling of His-Tagged Nanobodies with 99mTc: A Practical Guide.

[41] Pruszynski M, Koumarianou E, Vaidyanathan G, Revets H, Devoogdt N, Lahoutte T, Zalutsky MR (2013). Targeting breast carcinoma with radioiodinated anti-HER2 Nanobody. Nucl Med Biol.

[42] Vaneycken I, Devoogdt N, Van Gassen N, Vincke C, Xavier C, Wernery U, Muyldermans S, Lahoutte T, Caveliers V (2011). Preclinical screening of anti-HER2 nanobodies for molecular imaging of breast cancer. FASEB J.

[43] Mease RC, Foss CA, Pomper MG (2013). PET imaging in prostate cancer: focus on prostate-specific membrane antigen. Curr Top Med Chem.

[44] Keyaerts M, Xavier C, Heemskerk J, Devoogdt N, Everaert H, Ackaert C, Vanhoeij M, Duhoux FP, Gevaert T, Simon P, Schallier D, Fontaine C, Vaneycken I (2016). Phase I Study of 68Ga-HER2-Nanobody for PET/CT Assessment of HER2 Expression in Breast Carcinoma. J Nucl Med.

[45] Dijkers EC, Oude Munnink TH, Kosterink JG, Brouwers AH, Jager PL, de Jong JR, van Dongen GA, Schröder CP, Lub-de Hooge MN, de Vries EG (2010). Biodistribution of 89Zr-trastuzumab and PET imaging of HER2-positive lesions in patients with metastatic breast cancer. Clin Pharmacol Ther.

[46] Chakravarty R, Goel S, Cai W (2014). Nanobody: the “magic bullet” for molecular imaging?. Theranostics.

[47] Probst HC, McCoy K, Okazaki T, Honjo T, van den Broek M (2005). Resting dendritic cells induce peripheral CD8+ T cell tolerance through PD-1 and CTLA-4. Nat Immunol.

[48] Tsushima F, Yao S, Shin T, Flies A, Flies S, Xu H, Tamada K, Pardoll DM, Chen L (2007). Interaction between B7-H1 and PD-1 determines initiation and reversal of T-cell anergy. Blood.

[49] Van der Jeught K, Joe PT, Bialkowski L, Heirman C, Daszkiewicz L, Liechtenstein T, Escors D, Thielemans K, Breckpot K, Van der Jeught K, Joe PT, Bialkowski L, Heirman C (2014). Intratumoral administration of mRNA encoding a fusokine consisting of IFN-β and the ectodomain of the TGF-β receptor II potentiates antitumor immunity. Oncotarget.

[50] Spranger S, Spaapen RM, Zha Y, Williams J, Meng Y, Ha TT, Gajewski TF (2013). Up-regulation of PD-L1, IDO, and T(regs) in the melanoma tumor microenvironment is driven by CD8(+) T cells. Sci Transl Med.

[51] Su S, Hu B, Shao J, Shen B, Du J, Du Y, Zhou J, Yu L, Zhang L, Chen F, Sha H, Cheng L, Meng F (2016). CRISPR-Cas9 mediated efficient PD-1 disruption on human primary T cells from cancer patients. Sci Rep.

[52] Broisat A, Hernot S, Toczek J, De Vos J, Riou LM, Martin S, Ahmadi M, Thielens N, Wernery U, Caveliers V, Muyldermans S, Lahoutte T, Fagret D (2012). Nanobodies targeting mouse/human VCAM1 for the nuclear imaging of atherosclerotic lesions. Circ Res.

[53] Bradley ME, Dombrecht B, Manini J, Willis J, Vlerick D, De Taeye S, Van den Heede K, Roobrouck A, Grot E, Kent TC, Laeremans T, Steffensen S, Van Heeke G (2015). Potent and efficacious inhibition of CXCR2 signaling by biparatopic nanobodies combining two distinct modes of action. Mol Pharmacol.

[54] Conrath KE, Lauwereys M, Galleni M, Matagne A, Frère JM, Kinne J, Wyns L, Muyldermans S (2001). Beta-lactamase inhibitors derived from single-domain antibody fragments elicited in the camelidae. Antimicrob Agents Chemother.

[55] Lemaire M, D’Huyvetter M, Lahoutte T, Van Valckenborgh E, Menu E, De Bruyne E, Kronenberger P, Wernery U, Muyldermans S, Devoogdt N, Vanderkerken K (2014). Imaging and radioimmunotherapy of multiple myeloma with anti-idiotypic Nanobodies. Leukemia.

[56] Liechtenstein T, Perez-Janices N, Blanco-Luquin I, Goyvaerts C, Schwarze J, Dufait I, Lanna A, Ridder M, Guerrero-Setas D, Breckpot K, Escors D (2014). Anti-melanoma vaccines engineered to simultaneously modulate cytokine priming and silence PD-L1 characterized using ex vivo myeloid-derived suppressor cells as a readout of therapeutic efficacy. OncoImmunology.

[57] Goyvaerts C, Dingemans J, De Groeve K, Heirman C, Van Gulck E, Vanham G, De Baetselier P, Thielemans K, Raes G, Breckpot K (2013). Targeting of human antigen-presenting cell subsets. J Virol.

[58] Breckpot K, Dullaers M, Bonehill A, van Meirvenne S, Heirman C, de Greef C, van der Bruggen P, Thielemans K (2003). Lentivirally transduced dendritic cells as a tool for cancer immunotherapy. J Gene Med.

[59] Put S, Schoonooghe S, Devoogdt N, Schurgers E, Avau A, Mitera T, D’Huyvetter M, De Baetselier P, Raes G, Lahoutte T, Matthys P (2013). SPECT imaging of joint inflammation with Nanobodies targeting the macrophage mannose receptor in a mouse model for rheumatoid arthritis. J Nucl Med.

[60] Vanhove C, Defrise M, Bossuyt A, Lahoutte T (2009). Improved quantification in single-pinhole and multiple-pinhole SPECT using micro-CT information. Eur J Nucl Med Mol Imaging.

[61] Loening AM, Gambhir SS (2003). AMIDE: a free software tool for multimodality medical image analysis. Mol Imaging.

