# Supplementary material for: Non-invasive assessment of murine PD-L1 levels in syngeneic tumor models by nuclear imaging with nanobody tracers
Source: Oncotarget. 2017 Mar 30;8(26):41932–46. doi: 10.18632/oncotarget.16708 (PMC5522039; doi:10.18632/oncotarget.16708)
Supplement: Supplementary file 1 [file oncotarget-08-41932-s001.pdf]

Non-invasive assessment of murine PD-L1 levels in syngeneic tumor models by nuclear imaging with nanobody tracers

Supplementary Materials

```
     1        10        20        30         40        50         60         70        80        90        100        110                         120
     |********|*********|****** ***|******** *|*********|***** ****|***** ****|*********|*********|*********|**** *****|*1234567654321****** **|********
     <----- FR1-IMGT ---------> <CDR1-IMGT-> <- FR2-IMGT-----> <CDR2IMGT> <------------ FR3 -IMGT---------------> <-------- CDR3- IMGT-----> <FR4-IMGT->
A1   QVQLQESGG-GLVQPGGSLRLSCAAS GFTL----DYYA IGWFRQAPGKEREGVSC ISSS--DGST YYADSVK-GRFTISRDNAKNTVYLQMSSLKPEDTAVYYC GISGSCLL-----------EDYGMDY WGKGTQVTVSS
B1   QVQLQESGG-GLVHPGGSLRLSCAAS GFSL----DNYA IGWFRQAPGKEREGVSC ISSGS-EGRR YYADFVK-GRFTISRDNAKNTAFLQMNSLKPEDTADYYC ATVGFCSSQ--------YGMEFVGDY WGQGTQVTVSS
C1   QVQLQESGG-GLVQTGGSLRLSCAAS GSTV----SSSM MAWWRQTPGNQRELVAL VARG--NNTN YV-DSVK-GRFTVSRDNAKNTMYLQMNSLKPEDTAVYYC RILSV----------------NGIWY WGQGTQVTVSS
C2   QVQLQESGG-GLVQTGGSLRLSCAAS GSTV----SSSM MAWWRQTPGNQRELVAL VARG--NNTN YV-DSVK-GRFTVSRDNAKNTMYLQMNSLKPEDTAVYYC RILSV----------------NGIWY WGQGTQVTVSS
C3   QVQLQESGG-GLVQTGGSLRLSCAAS GSTV----SSSM MAWWRQTPGNQRELVAL VASG--NNTN YV-DSVK-GRFTVSRDNAKNTMYLQMNSLKPEDTAVYYC RILSV----------------NGIWY WGQGTQVTVSS
C4   QVQLQESGG-GLVQPGGSLRLSCAAS GSTV----SSSM MAWWRQTPGNQRELVAL VASG--NNTN YV-DSVK-GRFTVSRDNAKNTMYLQMNGLKPEDTAVYYC RLSVN-----------------GIWY WGQGTQVTVSS
C5   QVQLQESGG-GLVQAGGSLRLSCAAS GSTV----SSSM MAWWRQTPGNQRELVAL VASG--NNTN YV-DSVK-GRFTVSRDNAKNTMYLQMNSLKPEDTAVYYC RILSV----------------NGIWY WGQGTQVTVSS
C6   QVQLQESGG-GLVQAGDSLRLSCAAS GSTV----SSSM MAWWRQTPGNQRELVAL VASG--NNTN YV-DSVK-GRFTVSRDNAKNTMYLQMNSLKPEDTAVYYC RILSV----------------NGIWY WGQGTQVTVSS
C7   QVQLQESGG-GSVQAGESLTLSCTAS GSSL----SHKS VGWWRQTPGNQRELVAL VASG--NNTN YV-DSVK-GRFTVSRDNAKNTMYLQMNSLKPEDTAVYYC RILSV----------------NGIWY WGQGTQVTVSS
C8   QVQLQESGG-GLVQAGESLRLSCAAS GSTV----SSST MAWWRQTPGNQRELVAL VASG--GNTN YV-DSVK-GRFTVSRDNAKNTMYLQMNSLKPEDTAVYYC RILSM----------------NGIWY WGQGTQVTVSS
C9   QVQLQESGG-GLVQAGESLRLSCAGS GSTV----SSST MAWWRQTPGNQRELVAL VASG--GNTN YV-DSVK-GRFTVSRDNAKNTMYLQMNSLKPEDTAVYYC RILSM----------------NGIWY WGQGTQVTVSS
C10  QVQLQESGG-GLVQTGGSLRLSCAAS GSTV----SSST MAWWRQTPGNQRELVAL VASG--GNTN YV-DSVK-GRFTVSRDNAKNTMYLQMNSLKPEDTAVYYC RILSM----------------NGIWY WGQGTQVTVSS
C11  QVQLQESGG-GLVQTGGSLRLSCAAS GSTV----SSST MAWWRQTPGNQRELVAL VASG--GNTN YV-DSVK-GRFTVSRDNAKNTMYLQMNSLKPEDTAVYYC RILSV----------------NGIWY WGQGTQVTVSS
C12  QVQLQESGG-GLVQTGGSLRLSCAAS GSTV----SSST MAWWRQTPGNQRELVAL VASG--GNTN YV-DSVK-GRFTVSRDNAKNTMYLQMNSLKAEDTAVYYC RILSV----------------NGIWY WGQGTQVTVSS
C13  QVQLQESGG-GLVQTGGSLRLSCAAS GSTV----SSST MAWYRQTPGNQRDLVAL VGSG--GNIN YV-DSVK-GRFTVSIDNAKNTMYLQMNSLKPEDTAVYYC RILSV----------------NGIWY WGQGTQVTVSS
C14  QVQLQESGG-GLVQTGGSLRLSCAAS GSTV----GSST MAWWRQTPGNQRELVAL VGLG--GNTN YV-DSVK-GRFTVSIDNAENTMYLQMNSLKFEDTAVYYC RILSV----------------NGIWY WGQGTQVTVSS
C15  QVQLQESGG-GLVQTGGSLRVSCAVS GSTV----SRST MAWWRQTPGNQRELVAL VDSG--GKSN YV-DSVK-GRFTISTDNAKNTMYLQMNSLKPEDTAVYYC RILSV----------------QGIWY WGQGTQVTVSS
C16  QVQLQESGG-GLVQAGGSLKVSCAVS GSTV----SSST MAWWRQTPGNQRELVAL VDSG--GKSN YV-DSVK-GRFTISTDNAKNTMYLQMNSLKPEDTAVYYC RILSV----------------KGIWY WGQGTQVTVSS
D1   QVQLQESGG-GLVQAGGSLRLSCAAS GITV----SINA MAWYRQPPGKQRELVAL IIG----GST RYADSVK-GRFTISRDNAKNTVYLQMNSLKPEDTAVYAC NTR---------------------AY WGQGTQVTVSS
E1   QVQLQESGG-GLVQAGGSLRLSCATS GSIF----SIIS MGWYRQAPGKQRELVAL VFRG---GST VYADSVK-GRFTISGDIAKNTVYLQMDSLKPEDTAVYYC NVKSI----------------GTAQY WGQGTQVTVSS
E2   QVQLQESGG-GLVQAGGSLRLSCATS GSIF----SIIS MGWYRQAPGKQRELVAL VFRG---GST VYADSVK-GRFTISGDIAKNTVYLQMDSLKPEDTAVYYC NVKSI----------------GTAQY WGQGTQITVSS
E3   QVQLQESGG-GLVQPGGSLRLSCATS GSIF----SIIS MGWYRQAPGKQRELVAL VFRG---GST VYADSVK-GRFTISGDIAKNTVYLQMDSLKPEDTAVYYC NVKSI----------------GTAQY WGQGTQVTVSS
E4   QVQLQESGG-GLVHPGGSLRLSCATS GSIF----SIIS MGWYRQAPGKQRELVAL VFRG---GST VYADSVK-GRFTISGDIAKSTVYLQMDSLKPEDTAVYYC NAKPI----------------GTAQY WGQGTQVTVSS
E5   QVQLQESGG-GLVQPGGSLRLSCATS GSIF----SIIS MGWYRQAPGKQRELVAL VFRG---GST VYADSVK-GRFTISGDIAKSTVYLQMDSLKPEDTAVYYC NAKPI----------------GTAQY WGQGTQVTVSS
E6   QVQLQESGG-GLVQAGGSLRLSCATS GSIF----SIIS MGWYRQAPGKQRELVAL VFRG---GST VYADSVK-GRFTISGDIAKSTVYLQMDSLKPEDTAVYYC NAKPI----------------GTAQY WGQGTQVTVSS
F1   QVQLQESGG-GLVQAGGSLRLSCTAS RTTS----SINI MAWYRQAPGKRRELVAL SIRG---GNT HYADSVK-GRFTISGVHANNTVYLQMNSLKPEDTAVYYC NTG---------------------GY WGQGTQVTVSS
G1   QVQLQESGG-GLVQPGGSLRLSCAAS GSIS----SFNF MGWYRQAPGKQRELVAL STNS---GVT NVAGSVK-GRFTISRDNAKTTVYLQMNSLKPEDTAVYYC RVGAM-----------------GNLY WGQGTQVTVSS
G2   QVQLQESGG-GLVQPGGSLRLSCVAS GSIS----SFNF MGWYRQAPGKQRELVAL STNS---GVT NVAGSVK-GRFTISRDNAKTTVYLQMNSLKPEDTAVYYC RVGAM-----------------GNLY WGQGTQVTVSS
G3   QVQLQESGG-GLVQTGGSLRLSCAAS GSIS----SFNF MGWYRQAPGKQRELVAL STNS---GVT NVAGSVK-GRFTISRDNAKTTVYLQMNSLKPEDTAVYYC RVGAM-----------------GNLY WGQGTQVTVSS
G4   QVQLQESGG-GLVQAGGSLRLSCAAS GSIS----SFNF MGWYRQAPGKQRELVAL STNS---GVT NVAGSVK-GRFTISRDNAKTTVYLQMNSLKPEDTAVYYC RVGAM-----------------GNLY WGQGTQVTVSS
G5   QVQLQESGG-GLVQPGGSLRLSCAAS GSFF----RTYI VSWYRQAPGKRREAVAV MSNS---GNT NYADSVK-GRFTISRDNAVNTVYLQMNSLKPEDTAVYYC NLLKVT--------------VPPANY WGQGTQVTVSS
G6   QVQLQESGG-GLVQPGESLRLSCVAS GSFF----RTYI VSWYRQAPGKRREAVAV MSNS---GNT NYADSVK-GRFTISRDNAVNTVYLQMNSLKPEDTAVYYC NLLKVT--------------VPPANY WGQGTQVTVSS
G7   QVQLQESGG-GLVQSGGSLRLSCAAS GSFF----RTYI VSWYRQAPGKRREAVAV MSNS---GNT NYADSVK-GRFTISRDNAVNTVYLQMNSLKPEDTAVYYC NLLKVT--------------VPPANY WGQGTQVTVSS
H1   QVQLQESGG-GLVQAGGSLRLSCAAS GRTF----SSRG MGWFRQAPGKEREFVAA ISWG--GGTT YYADSVK-GRFTISRDNAKNTVYLQMNSLKPEDTAVYYC AAHTGF--------------PHEYDY WGQGTQVTVSS
I1   QVQLQESGG-GLVQAGGSLRLSCAAS GRTF----RRYD MGWFRQAPGKEREFVAA STWS--ADDT YYAGSVK-GRFTISRDNAKNTVYLQMNSLKPEDTAVYYC AAGETRKYGF-------RTTEGYYDS WGQGTQVTVSS
L1   QVQ-----------AGGSLRLSCAAS GYRY----CRYD MSWYRQAPGKEREFVSA IDSY---GSP TYADTVL-GRFTISRDNAKNTVYLQMNSLKAEDTAMYYC KIDSTGWS-----------SRCGKTY WGQGTQVTVSS
M1   QVQ-----------AGDSLRLACTLF KYPT----NNLC LAWFRQAPGKEREGVAA LNTD--GVGT YYAKSVE-GRFTISQDKSKNEGYLQMDNLKPEDTAIYYC AADPTSRCNNYVQGRWWHEIGSRVWD WGQGTQVTVSS
```

Supplementary Figure 1: Selection of anti-mouse-PD-L1 specific Nbs. Amino acid sequence alignment of 37 Nbs binding PD-L1. The Nb sequence includes three complementarity-determining regions (CDR 1, 2, 3; indicated in red) and four framework regions (FR1-4, indicated in black). FRs are relatively conserved but CDRs vary widely among Nbs.

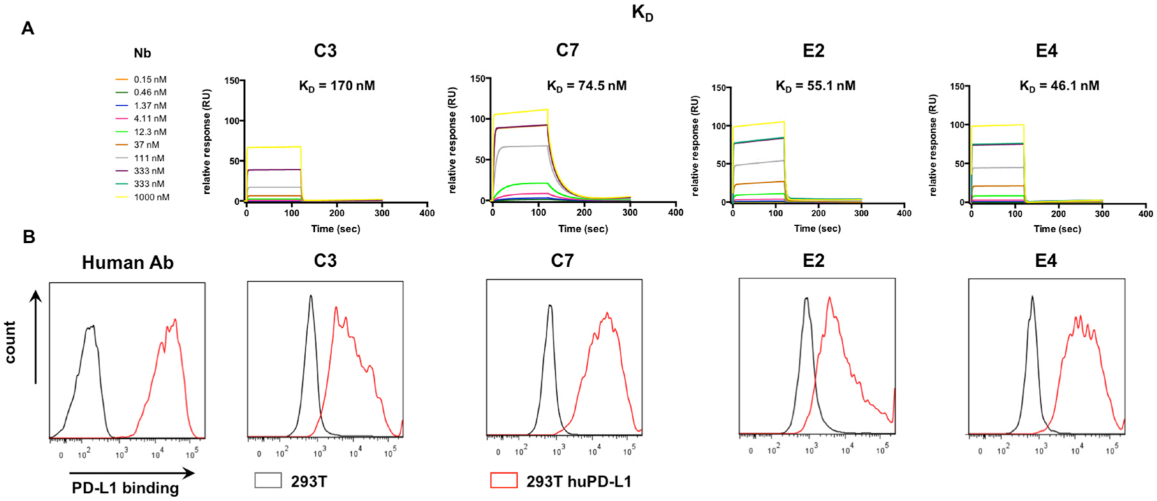

Supplementary Figure 2: Summary of the affinity for human PD-L1 of purified anti-PD-L1 Nbs C3, C7, E4 and E2. (**A**) Affinity/kinetics SPR study of purified Nbs interacting with immobilized His-tagged recombinant human PD-L1 protein. Sensorgrams of different concentrations of the Nbs are shown (n = 1). (**B**) Representative flow cytometry results, showing staining of unmodified HEK293T cells (grey line) or HEK293T cells lentivirally modified to express human PD-L1 (293T huPD-L1, red line) with mAbs specific for human PD-L1 or Nbs C3, C7, E4 and E2 (n = 3).

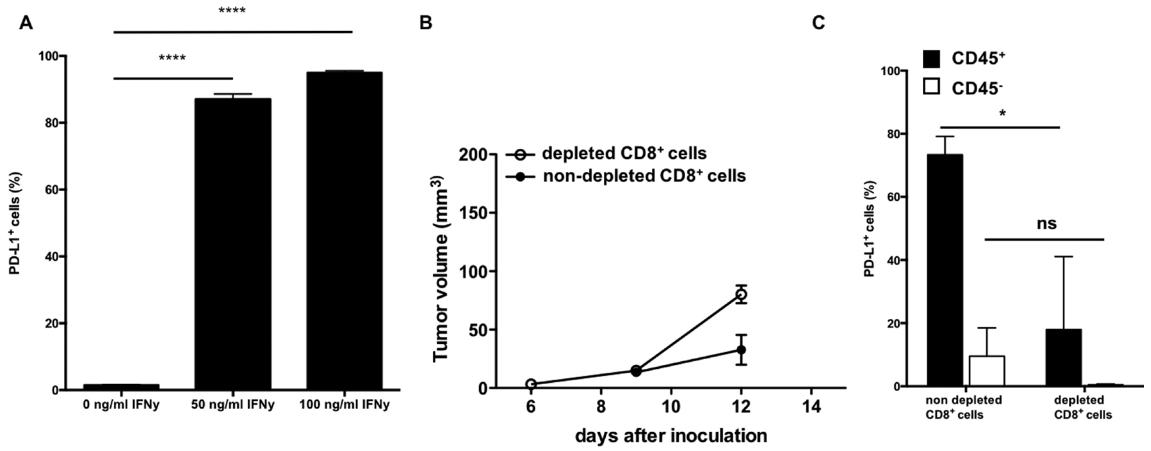

Supplementary Figure 3: (**A**) TC-1 cells modified with shRNA targeting mouse PD-L1 (knock-down, KD) were left untreated or treated with 50 or 100 ng/ml recombinant mouse IFN-γ. Flow cytometry was performed to measure the percentage of TC-1 cells that express PD-L1 (n = 3). (**B**–**C**) PD-L1 KD TC-1 cells were injected subcutaneously at the tail base of wild type mice, which were pre-treated with a CD8+ depleting antibody (depleted CD8+ cells) or an isotype matched control antibody (non-depleted CD8+ cells). (**B**) Tumor growth was measured every other day. The tumor size in function of time is shown as mean ± SEM. (**C**) Tumors were isolated on day 12 to evaluate the expression of PD-L1 on tumor cells (CD45−, white bar) and tumor-infiltrating immune cells (CD45+, black bar) in flow cytometry. The graph summarizes the percentage of PD-L1 expressing cells as mean ± SEM (n = 3 for non CD8-depleted mice and n = 4 for CD8-depleted mice).

Supplementary Table 1: Characterization of the periplasmic extracts of 37 anti-PD-L1 Nbs

						Flow cytometry (MFI)			
	ELISA (OD-value)			Biacore (affinity)		Human		Mouse	
Nb	Mouse	Human	No Ag	Mouse	Human	PD-L1+	PD-L1−	PD-L1+	PD-L1−
A1	2.5794	0.0945	0.0968	no signal	23 nM	10174	699	116	129
B1	0.897	0.2141	0.195	123 nM	6 nM	716	688	-56	-45
C1	3.6802	0.4291	0.1554	327 pM	143 nM	14251	1254	1101	215
C2	3.743	0.4727	0.1838	ND	ND	ND	ND	ND	ND
C3	3.7828	1.3394	0.1525	140 pM	32 nM	3315	272	431	36
C4	3.7723	0.8755	0.1812	187 pM	342 nM	10792	348	581	43
C5	3.6962	0.8694	0.1243	ND	ND	ND	ND	ND	ND
C6	3.7024	0.6344	0.1532	289 pM	94 nM	7528	691	770	208
C7	3.7739	1.1077	0.3419	3.1 nM	27 nM	25555	2300	275	-4
C8	3.7573	1.1562	0.1777	ND	ND	ND	ND	ND	ND
C9	3.6941	0.7949	0.1238	2.2 nM	44 nM	5050	515	461	234
C10	3.7161	0.6557	0.2129	6.7 nM	141 nM	20856	572	452	98
C11	3.8662	0.5572	0.2175	12 nM	246 nM	27267	577	378	105
C12	3.5804	0.4049	0.0917	11.4 nM	181 nM	19746	1116	348	-46
C13	3.5369	1.0144	0.1249	33 nM	162 nM	26347	2671	36	-8
C14	3.6701	0.7512	0.0911	1.8 nM	71 nM	8650	1350	96	91
C15	3.6368	0.4461	0.1504	6.5 nM	335 nM	13530	684	27	-51
C16	3.6768	0.5203	0.1853	15 nM	520 nM	15277	411	202	142
D1	3.6391	2.8148	2.4878	12 nM	291 nM	3054	506	196	-72
E1	3.6391	2.8148	2.4878	2.3 nM	12 nM	4944	681	135	-29
E2	2.9933	1.0964	0.5687	5.6 nM	94 nM	1301	34	283	119
E3	3.9717	3.0705	1.7095	ND	ND	1893	429	-46	-62
E4	3.9304	3.0665	1.0478	ND	ND	5110	259	69	64
E5	2.8355	0.1141	0.0989	14 nM	54 nM	1704	454	37	-7
E6	2.4418	0.6909	0.3211	6.9 nM	217 nM	2348	500	-54	-20
F1	3.6697	0.4092	0.2388	ND	ND	ND	ND	ND	ND
G1	3.5102	0.3395	0.1831	4.4 nM	50 nM	2795	616	120	-5
G2	4	2.3125	1.4331	5.5 nM	158 nM	1936	423	341	43
G3	3.7986	1.6653	0.77	5.0 nM	907 nM	1543	699	55	-46
G4	3.892	1.3798	0.7293	6.2 nM	74 nM	822	305	227	88
G5	3.9486	3.5717	2.5806	3.9 nM	16 nM	3357	336	289	-29
G6	4	3.7483	2.912	5.0 nM	21 nM	5902	8522	-47	21
G7	1.0341	0.3335	0.2443	3.4 nM	129 nM	2870	159	319	141
H1	3.7375	0.8713	0.6773	ND	ND	ND	ND	ND	ND
I1	3.5587	0.2076	0.2332	67 nM	322 nM	3697	2309	28	-35
L1	2.307	ND	0.899	15 nM	34 nM	ND	ND	ND	ND
M1	ND	5.975	0.254	13 nM	17 nM	ND	ND	ND	ND

Summary of the binding of the Nbs to mouse or human PD-L1 using ELISA, the affinity of the Nbs for mouse and human PD-L1 using SPR and the binding of the Nbs to cells expressing mouse (B16) or human (293T) PD-L1 using flow cytometry.
MFI, Mean Fluorescence Intensity; ND, not determined.

Supplementary Table 2: Biodistribution of 99mTc-labeled Nbs C3, C7, E4 and E2 in wild type (WT) or PD-L1-deficient (KO) mice 80 minutes after intravenous administration. Data are expressed as mean and standard deviation (SD) of percentage of injected activity per gram of tissue (%IA/g) (*n* = 3). See Supplementary_Table_2

Supplementary Table 3: Tissue versus blood (T/B) and anti-PD-L1 Nb versus control (ctrl) Nb uptake ratios in wild type (WT) mice, and tissue uptake ratios in WT versus PD-L1-deficient (KO) mice 80 minutes after intravenous administration of ^{99m}Tc-labeled Nbs C3, C7, E4 and E2. Ratios are calculated from the data presented in supplementary table 2 (*n* = 3). See Supplementary_Table_3

Supplementary Table 4: Biodistribution of ^{99m}Tc-labeled Nbs C3 and E2 injected in wild type (WT) mice bearing PD-L1 knock-down (KD) or PD-L1 knock-in (KI) cells (left). Biodistribution of 99mTc-labeled Nbs C3 and E2 injected in wild type (WT) mice bearing wild type (WT) TC-1 cells or injected in CD8+-depleted PD-L1 KO (KO) mice bearing PD-L1 knock-out (KO) cells (right), 80 minutes after intravenous administration. Data are expressed as mean and standard deviation (SD) of percentage of injected activity per gram of tissue (%IA/g) (*n* = 6). See Supplementary_Table_4

Supplementary Table 5: Tissue-to-blood uptake ratios of 99mTc-labeled Nbs C3 and E2 injected in wild type (WT) mice bearing PD-L1 knock-down (KD) or PD-L1 knock-in (KI) cells (left)

	shRNA-modified TC-1 model						CRISPR/Cas9-modified TC-1 model					
**Tumor**	**KD**			**KI**			**WT**			**KO**		
**Mice**	**WT**			**WT**			**WT**			**KO**		
	Ctrl Nb	C3	E2	Ctrl Nb	C3	E2	Ctrl Nb	C3	E2	Ctrl Nb	C3	E2
**thymus**	0,3	3,8	3,4	0,3	3,6	3,6	0,3	2,2	2,6	0,3	0,2	0,4
**heart**	0,4	4,1	4,0	0,4	3,7	1,8	0,4	2,5	1,4	0,4	0,4	0,5
**lungs**	0,7	7,1	6,4	0,8	6,4	3,3	0,7	3,9	2,5	0,9	0,8	1,2
**liver**	0,6	13,7	3,2	0,9	10,4	2,7	0,5	7,7	2,5	0,7	3,8	1,7
**spleen**	0,3	16,3	6,0	0,4	18,5	7,3	0,3	8,9	4,5	0,4	1,1	0,6
**pancreas**	0,2	1,6	1,1	0,2	1,5	0,9	0,2	1,1	0,6	0,3	0,2	0,3
**kidneys**	281,5	281,1	265,5	293,3	302,7	284,9	142,1	90,3	159,3	323,7	117,9	319,4
**Stomach**	0,7	2,5	2,1	0,6	2,2	1,5	0,5	1,5	0,8	0,6	0,4	0,6
**small intestines**	0,6	7,0	3,6	0,4	6,8	3,2	0,3	4,3	1,9	0,7	0,6	0,7
**large intestines**	0,4	3,3	2,0	0,4	3,2	1,9	0,3	2,3	2,1	0,4	0,3	0,5
**muscle**	0,3	0,9	0,8	0,2	0,8	0,6	0,1	0,5	0,3	0,2	0,1	0,2
**bone**	0,3	1,5	0,8	0,3	1,1	0,8	0,2	0,8	0,4	0,2	0,3	0,4
**lymph nodes**	0,4	6,8	3,3	0,7	6,2	3,7	0,4	2,7	2,1	0,9	0,3	0,6
**white fat**	0,2	1,4	1,2	0,2	1,5	0,9	0,2	0,9	0,7	0,2	0,1	0,2
**brown fat**	0,3	8,7	6,4	0,3	6,7	5,9	0,3	4,8	5,2	0,4	0,2	0,5
**tumor**	0,8	6,6	8,1	0,8	5,2	3,4	0,4	1,4	1,0	1,1	0,7	1,5
**blood**	1,0	1,0	1,0	1,0	1,0	1,0	1,0	1,0	1,0	1,0	1,0	1,0

Tissue –to-blood uptake ratios of ^{99m}Tc-labeled Nbs C3 and E2 injected in wild type (WT) mice bearing wild type (WT) TC-1 cells or CD8+-depleted PD-L1 KO (KO) mice bearing PD-L1 knock-out (KO) cells (right), 80 minutes after intravenous administration of ^{99m}Tc-labeled Nbs C3 and E2. Ratios are calculated from the data presented in Supplementary Table 4 (*n* = 6).
